# A Non-Toxic Binuclear Vanadium(IV) Complex as Insulin Adjuvant Improves the Glycemic Control in Streptozotocin-Induced Diabetic Rats

**DOI:** 10.3390/ph17040486

**Published:** 2024-04-11

**Authors:** Mateus S. Lopes, Gabriel B. Baptistella, Giovana G. Nunes, Matheus V. Ferreira, Joice Maria Cunha, Kauê Marcel de Oliveira, Alexandra Acco, Maria Luiza C. Lopes, Alexessander Couto Alves, Glaucio Valdameri, Vivian R. Moure, Geraldo Picheth, Graciele C. M. Manica, Fabiane G. M. Rego

**Affiliations:** 1 Post-Graduation Program in Pharmaceutical Sciences, Federal University of Paraná, Curitiba 80210-170, PR, Brazil; mateus.santana@ufpr.br (M.S.L.); clarindomarialuiza@gmail.com (M.L.C.L.); gvaldameri@ufpr.br (G.V.); vivian.moure@ufpr.br (V.R.M.); gpicheth@ufpr.br (G.P.); 2 Department of Chemistry, Federal University of Paraná, Curitiba 81531-980, PR, Brazil; gabriel.baptistella@ufpr.br (G.B.B.); nunesgg@ufpr.br (G.G.N.); 3 Post-Graduation Program in Pharmacology, Federal University of Paraná, Curitiba 81531-980, PR, Brazil; matheus.ferreira1@ufpr.br (M.V.F.); joice.cunha@ufpr.br (J.M.C.); kaue.marcel@ufpr.br (K.M.d.O.); aleacco@ufpr.br (A.A.); 4 School of Biosciences and Medicine, Faculty of Health and Medical Sciences, University of Surrey, Guildford GU2 7XH, UK; a.coutoalves@surrey.ac.uk; 5 Department of Bioscience One Health of Federal University of Santa Catarina, Curitibanos 88520-000, SC, Brazil; graciele.manica@ufsc.br

**Keywords:** oxidovanadium(IV), vanadium, STZ-induced diabetic rats, adjunctive to insulin therapies, treatment efficacy

## Abstract

Diabetes mellitus (DM) complications are a burden to health care systems due to the associated consequences of poor glycemic control and the side effects of insulin therapy. Recently. adjuvant therapies, such as vanadium compounds, have gained attention due to their potential to improve glucose homeostasis in patients with diabetes. In order to determine the anti-diabetic and antioxidant effects of the oxidovanadium(IV) complex (Et_3_NH)_2_[{VO(OH}_2_)(ox)_2_(µ–ox)] or Vox2), rats with streptozotocin (STZ)-induced diabetes were treated with 30 and 100 mg/kg of Vox2, orally administered for 12 days. Vox2 at 100 mg/kg in association with insulin caused a 3.4 times decrease in blood glucose in STZ rats (424 mg/dL), reaching concentrations similar to those in the normoglycemic animals (126 mg/dL). Compared to insulin alone, the association with Vox2 caused an additional decrease in blood glucose of 39% and 65% at 30 and 100 mg/kg, respectively, and an increased pancreatic GSH levels 2.5 times. Vox2 alone did not cause gastrointestinal discomfort, diarrhea, and hepatic or renal toxicity and was not associated with changes in blood glucose level, lipid profile, or kidney or liver function. Our results highlight the potential of Vox2 in association with insulin in treating diabetes.

## 1. Introduction

Diabetes mellitus (DM) is a global pandemic that has significantly contributed to the escalating mortality associated with non-communicable diseases [1,2,3]. DM is an intricate and persistent medical condition characterized by elevated glycemia levels and carbohydrate and lipid metabolism disorders. These disturbances give rise to microvascular complications that affect the retina, renal glomeruli, and peripheral nerves [4,5,6,7]. Upon the onset of hyperglycemia, individuals with any form of diabetes face a common risk of developing chronic complications, albeit with varying rates of progression. Individuals with type 1 and long-standing type 2 diabetes require insulin to regulate blood glucose levels, which is essential for their survival [4,6,7].

Prolonged insulin therapy may lead to various undesirable outcomes, including lipodystrophy, increased body weight, and hypoglycemia [8]. Consequently, identifying novel agents that mimic or enhance the effect of insulin on oral consumption is crucial for improving diabetes treatment [9].

The first report describing vanadium compounds in treating diabetes dates back to 1899. Nevertheless, in the last two decades, there has been a noteworthy relationship in pharmaceutical research regarding vanadium compounds [10]. A timeline of events relevant to vanadium use in diabetes is shown in Supplementary Figure S1. Most recent studies have focused on insulin-like and insulin-mimetic properties [11,12,13,14,15]. Favorable outcomes have been observed upon application of various categories of vanadium complexes in diabetic models, both in vitro and in vivo. Furthermore, these complexes exhibited insulin-enhancing activity [12,13,14,16,17]. The beneficial effects of vanadium in reducing hyperglycemia have been reported in experimental and clinical trials in humans [18,19].

Although these findings are promising, some potential toxic effects and water solubility problems are associated with vanadium salts [20,21,22,23,24]. The use of organic vanadium complexes has been explored to mitigate these issues, and these compounds induce glucose-lowering effects at a significantly lower dose than that previously used for inorganic vanadium salts, without any apparent toxicity [25,26,27].

These compounds have been linked to notable enhancements in glucose absorption, restraint of free fatty acid release, and prevention of oxidative damage caused by diabetes [12,17,28]. The fact that the new vanadium compounds also reduce diabetes-related oxidative stress (antioxidant effect) further increases their value [29,30,31].

In this context, vanadium has attracted attention as a potential adjunct treatment for diabetes [32]. Numerous researchers have conducted experiments to address issues related to the continuous application of vanadium compounds and their resultant accumulation in tissues, which may result in notable side effects [20,21,33]. Investigators have explored the utilization of vanadium compounds in the configuration of metal-ion chelates to address these concerns [11,13,32].

Various ligands have been used to overcome these side effects and enhance vanadium absorption through the gastrointestinal tract [34]. Many VO^2+^ chelates have been synthesized using multifarious ligands [35]. The preferred ligands were 3-hydroxy-4-pyran (maltol) [26,36,37,38], kojic acid [39], picolinic acid [40,41,42,43], biguanide [44], acetylaceton [45,46], and imidazole derivatives [47].

**Figure 1:**
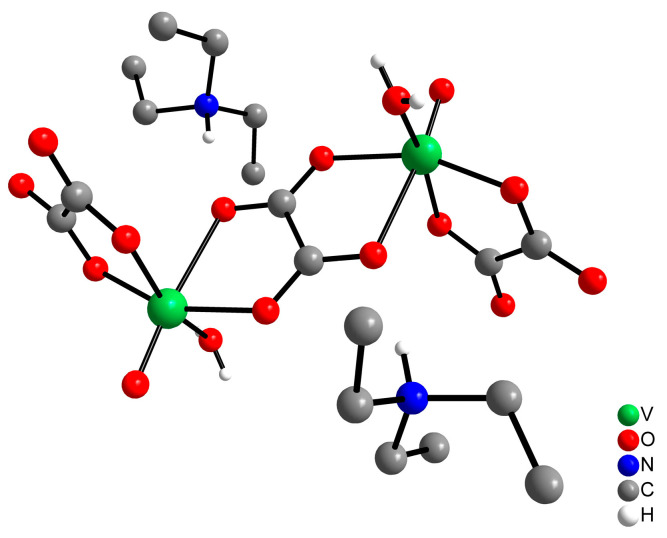
Ball and stick representation of the (Et_3_NH)_2_[{VO(OH}_2_)(ox)_2_(µ–ox)], or Vox2, complex. The Vox2 structure consists of an anionic binuclear complex formulated as [V(O)(OH_2_)(ox)_2_(μ-ox)]^2−^, with two triethylammonium (Et3NH) as counter ions. A bis-bidentate oxalate ligand bridges the two six-coordinate vanadium centers, and the coordination sphere of each metal ion is completed by three terminal ligands: one bidentate oxalate (ox^2−^), one water molecule, and an oxide group (O^2−^). Molecular mass = 638.37 g mol^−1^.

Recently, our group described the synthesis and characterization of (Et_3_NH)_2_[{VO(OH}_2_)(ox)_2_(µ–ox)] (Vox2, Figure 1), a centrosymmetric oxidovandadium(IV) binuclear complex containing the bioligand oxalate (ox^2−^) [48]. Treatment of human hepatocellular carcinoma (HepG2) cells with this complex in a hyperglycemic medium showed a similar or better response to the uptake of 2-NBDG (2-[N-(7-nitrobenz-2-oxa-1,3-diazol-4-yl)amino]-2-deoxy-D-glucose), a fluorescent glucose analog, than with insulin at concentrations of vanadium below the toxicity threshold. Moreover, the previous stability studies in aqueous solutions have shown that the binuclear structure of Vox2 is maintained even at low concentrations, suggesting that this species could be promising for further anti-diabetic studies in vivo [48]. To achieve this, we used streptozotocin (STZ)-induced diabetic rats to evaluate the effect of Vox2 on glucose metabolism in the presence and absence of insulin and observed a remarkable reduction in blood glucose levels. We believe that these findings shed light on the insulin-mimetic activity of oxidovanadium(IV) complexes, especially those with water-soluble oxalate species.

## 2. Results

### 2.1. Preparation of the Oxidovanadium(IV) Complex

To ensure that the synthesized compound was the same compound used by Baptistella et al. [48], confirmatory tests were carried out. The original synthesis was carried out by scaling the amounts of reactants and solvent 10-fold to produce sufficient amounts of Vox2 to perform the in vivo studies. The powder X-ray diffraction pattern of the light greenish-blue crystals was in good agreement with that generated from the previously described single-crystal X-ray diffraction structure [48] (Figure S2), confirming the purity of the compound in bulk. The structure of the [V^IV^(O)(OH_2_)(ox)_2_(μ-ox)]^2–^ anion comprises a binuclear complex bridged by a bis-bidentate oxalate ligand, with the bidentate oxalate and one water molecule coordinated to each oxidovanadium(IV) center (Figure 1). Electron paramagnetic resonance (EPR) analysis of aqueous solutions of Vox2 at 0.1 and 1.0 mmol/L showed a broad line (g = 1.986 and Δ_p-p_ = 23 mT), as expected for a binuclear vanadium(IV) species with magnetic interaction between the metal centers (Figure S3). The stability study of Vox2 in aqueous solution in a range of concentrations and in Dulbecco modification of minimum essential media (DMEM) over a period of 24 h was previously described [48].

### 2.2. Vox2 Shows In Vitro Antioxidant Activity

Previous to the evaluation of oxidative stress in vivo (liver and pancreas), the antioxidant effect of Vox2 per se was evaluated in vitro, in a cell-free system, using 2,2-diphenyl-1-picrylhydrazyl (DPPH) free-radical scavenging activity and the ferric-reducing antioxidant power (FRAP) methods. Vox2 showed antioxidant activity in both tests, nevertheless with different sensitivities. In the DPPH test (Figure S4A), Vox2 showed antioxidant activity at lower concentrations, between 1 and 100 µg/mL, similarly to the positive control (AA; ascorbic acid), which scavenged the free radical DPPH at 50 µg/mL. The bluish color of the Vox2 may have interfered with the absorbance, reducing the effectiveness of the test in higher concentrations. On the other hand, using the FRAP method (Figure S4B), the higher the absorbance of the sample, the greater the antioxidant potential, and this effect was obtained with Vox2 at elevated concentrations, between 30 and 1000 µg/mL. Both tests showed that the molecule of Vox2 has antioxidant potential.

### 2.3. Evaluation of the Acute Toxic Effects of the Vox2 Administration

Rodent behavioral assays were first conducted to gain insights into acute toxic effects of Vox2. The open-field test allows the detection of some effects, such as sedation, hypo/hyperactivity, and anxiety [49]. The animals not previously subjected to experimentation (also described as Naïve) were divided into 3 groups: orally treated with 30 mg/kg (0.0469 mmol/kg) of Vox2 (Naïve V_30_), with 100 mg/kg (0.156 mmol/kg) of Vox2 (Naïve V_100_), and vehicle (Naïve), and 1 h after the treatments, they were submitted to this test. During this period, the animals were freely offered food, kaolin (white clay used to evaluate the emetic response in rats), and water.

**Figure 2:**
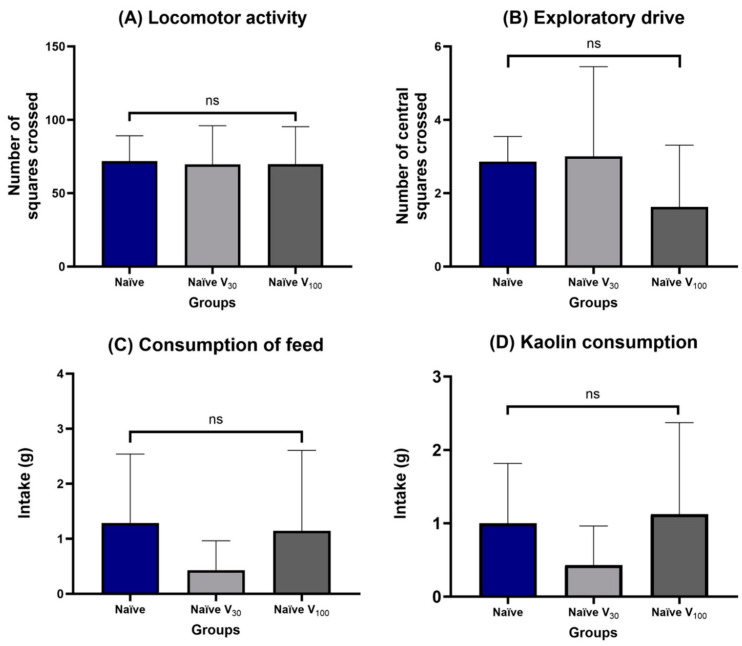
Open-Field Test after Vox2 administration.

Comparisons using ANOVA of locomotor activity (*p* = 0.545), exploratory drive (*p* = 0.585), food (*p* = 0.321), and kaolin (*p* = 0.517) intake after Vox2 administration showed no difference among the groups throughout the study (Figure 2A,B,C and D, respectively). Exposure to Vox2 did not affect any of the parameters evaluated in the open-field test (Figure 2).

Values are mean ± SD. Locomotor activity for the number of squares crossed (A), exploratory drive for the number of central squares crossed (B), food intake (C), and kaolin intake (D) were measured for the Naïve group (n = 7), control group treated with vehicle; Naïve V_30_ (n = 7), animals treated with 30 mg/kg Vox2; and Naïve V_100_ (n = 8), animals treated with 100 mg/kg Vox2. All the animals were used once. Probability was measured using one-way ANOVA with a significance of *p* > 0.05, or non-significant (ns).

### 2.4. Vanadium Compound Effects in Diabetes

The in vivo experiments assessed the effects of Vox2 with and without insulin on diabetic (DM) and non-diabetic (NG) animals using standard insulin treatment as a baseline (Figure S5).

**Figure 3:**
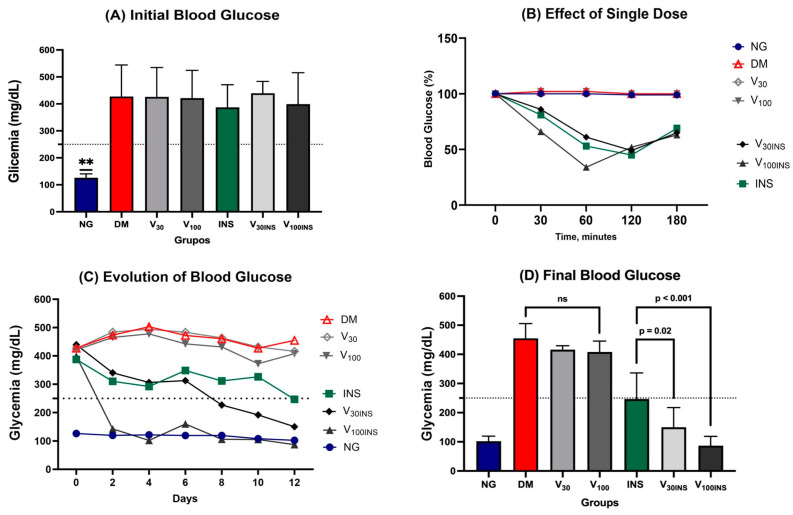
Effect of Vox2 on blood glucose concentration in the different experimental stages. Values are mean ± SD (mg/dL) on first (**A**) and last (**D**) days of treatment. Blood glucose % (**B**) at 0, 30, 60, 120, and 180 min after treatment was initiated, assuming 100% of the concentration at time zero (0), collected immediately before the start of treatment. Mean glycemia measurement during the study period (**C**). Groups are NG (n = 10), normoglycemic and non-diabetic control group; Group DM (n = 6), diabetes mellitus group, STZ-induced diabetic rats untreated; Group INS (n = 6), induced diabetic rats treated with insulin; Group V_30_ (n = 6), induced diabetic rats treated with 30 mg/kg of Vox2; Group V_100_ (n = 6), induced diabetic rats treated with 100 mg/kg of Vox2, Group V_30INS_ (n = 6), induced diabetic rats treated with insulin plus 30 mg/kg of Vox2 and Group V_100INS_ (n = 6), induced diabetic rats treated with insulin plus 100 mg/kg of Vox2. The dotted line corresponds to a glucose concentration of 250 mg/dL, which is an established criterion for diabetes. In (**A**), all groups are not different except for the control group (NG), which is smaller than the others (**, *p* < 0.001). In (**C**), groups DM, V_30_, and V_100_ were not different. Groups NG, V_30INS_, and V_100INS_ were not different. Statistical analysis was performed using a one-way ANOVA followed by Tukey’s test.

Diabetes (DM group) was consolidated after STZ administration (Figure 3A), increasing blood glucose levels above the established criteria (>250 mg/dL or >13.9 mmol/L). STZ rats showed mean glycemia >400 mg/dL (>22.2 mmol/L), a concentration 3.4 times higher than that for the control group (NG) (respectively, 424 ± 98 vs. 126 ± 14 mg/dL, *p* < 0.001).

Once diabetes was successfully established, the treatments were initiated with insulin subcutaneous injections (INS), 30 and 100 mg/kg oral doses of Vox2 (V_30_ and V_100_), and the combination of insulin with Vox2 (V_30INS_ and V_100INS_). On the first day, glycemia was monitored in animals at 30, 60, 120, and 180 min after the administration of the 5 different treatments (Figure 3B). The samples collected before the treatment (time 0) were considered as 100%. The groups that received insulin (INS, V_30INS_, and V_100INS_) showed a reduction in blood glucose mean levels by approximately 50% at 60 (INS = 190 mg/dL, V_30INS_ = 256 mg/dL, and V_100INS_ = 147 mg/dL) and 120 min (INS = 169 mg/dL, V_30INS_ = 207 mg/dL, and V_100INS_ = 224 mg/dL) compared to their concentrations in time 0 (INS = 370 mg/dL, V_30INS_ = 419 mg/dL, and V_100INS_ = 428 mg/dL). The groups that did not receive insulin showed no differences (DM = 453 mg/dL; V_30_ = 442 mg/dL; and V_100_ = 456 mg/dL).

Blood glucose was monitored every 2 days during treatment (12 days), and the values are shown in Figure 3C. Time zero was considered to be 4 days after STZ induction of diabetes. The DM, V_30_, and V_100_ groups did not differ throughout the 12 days of treatment. However, the V_30INS,_ V_100INS_, and INS groups had lower blood glucose levels than the DM group throughout the study period. V_30INS_ slowly reduced blood glucose until reaching a concentration of 150 mg/dL on day 12, a reduction of 67% compared to the DM group. Interestingly, the V_100INS_ group showed decreased glucose to normoglycemic levels (defined as a mean glucose concentration for the NG group of 117 mg/dL) within the first 72 h and remained stable until the end of treatment, a reduction of 81% compared to the DM. Vox2 associated with insulin suggests a dose-dependent action (Figure 3C).

Considering only the last day of the experiment, Vox2 alone did not cause any changes in blood glucose levels at concentrations of 30 and 100 mg/kg (V_30_ and V_100_ groups, respectively) (Figure 3D). In contrast, Vox2 associated with insulin showed a decrease in glycemia of 39% or 1.6-fold (150.0 ± 67.3 mg/dL) with a dose of 30 mg/kg (V_30INS_) and 65% or 2.8-fold (86.8 ± 32.0 mg/dL) for 100 mg/kg (V_100INS_) when compared to treatment with insulin alone (INS; 246 ± 89.7 mg/dL) (Figure 3D). That of the NG control group (102.0 ± 17.4 mg/dL) did not differ from that of the V_30INS_ (*p* = 0.609) or V_100INS_ (*p* = 0.998) groups.

Taking the results together, Figure 3D shows that Vox2 alone did not have a hypoglycemic effect; the diabetic group (DM) and diabetic group treated with Vox2 alone at 30 mg/kg (V_30_) and 100 mg/kg (V_100_) presented similar glucose concentrations (respectively, 455 mg/dL, 416 mg/dL, and 408 mg/dL, *p* > 0.05). Nonetheless, the association with insulin promoted enhancement of the action of this hypoglycemic agent in 39% (V_30INS_ = 150 mg/dL vs. INS = 247 mg/dL) and 65% (V_100INS_ = 87 mg/dL vs. INS = 247 mg/dL). At the concentrations used, the decrease in glycemia with Vox2 associated with insulin promoted effective glycemic control, superior to that of insulin alone (INS = 247 mg/dL) and similar to that of the control animals (NG = 102 mg/dL) in the experimental period.

### 2.5. General Clinical Observations and Biochemical Markers

To evaluate the effects of Vox2 on metabolism and tissues affected by diabetes, the glycemic, lipid, and nutritional profiles, as well as the liver and kidney function, were determined at the end of the experimental period. During the experimental period, the bodyweight and water, kaolin, and food consumption were monitored.

The levels of biomarkers, organ mass, and nutritional parameters in all animal groups at the end of the experiment (12 days) are summarized in Table 1. The average concentration of biomarkers in each group were compared to that of the control (NG) or diabetic (DM) groups, considering these groups as 100%.

The lipid profile showed an increase of approximately 50–70% in total cholesterol in the DM, V_30_, and V_100_ groups when compared to the control (NG). The groups that received insulin, INS, V_30INS_, and V_100INS_ showed no differences from the NG group. A similar pattern was observed for HDL cholesterol, suggesting that the induction of diabetes was responsible for these changes and that the presence of Vox2 was not relevant. Triglycerides did not show variations among the groups (Table 1).

Renal filtration function was evaluated using urea and creatinine markers. Urea increased by approximately 50 to 60% in all diabetic groups when compared to the control group (NG). Creatinine, a more specific marker of renal function, showed no difference between the groups (ANOVA, *p* = 0.237), a pattern similar to that observed for uric acid (Table 1).

The animals in all groups maintained a stable and normal nutritional pattern, as indicated by the absence of differences in the concentrations of total protein and albumin (Table 1). The stability of these markers suggests that the hepatic and renal systems were intact during the experimental period.

Hepatocellular damage marker enzymes, including alanine transaminase (ALT) and aspartate transaminase (AST), did not differ between the groups, as did lactate dehydrogenase (LDH), a ubiquitous enzyme. Alkaline phosphatase (ALP), a marker of cholestasis and bone damage, did not differ among the NG, DM, INS, V_30_, V_100_, and V_30INS_ groups, corroborating other enzymatic results of the absence of acute injury in the target tissues of this marker. ALP in the V_100INS_ group presented 32% lower activity than the INS group. These results corroborate the absence of hepatic histological alteration (Figure S6). No hepatocyte congestion, inflammatory infiltrate, fibrosis, and vacuolization or any unfavorable changes were observed in the studied tissue sections, indicating that Vox2 is not hepatotoxic.

Amylase activity was reduced by approximately 50–60% when the control group (NG) was compared with the groups that did not receive insulin, such as DM, V_30_, and V_100_. The presence of insulin in (INS, V_30INS_, and V_100INS_) reestablished was similar to that in the control group (NG) (Table 1).

Oxidative stress markers in the liver (reduced glutathione, GSH, lipid peroxidation, LPO, and total tissue proteins) showed no differences among the groups (Table 1). In contrast, treatment with 30 mg/kg and 100 mg/kg of Vox2 associated with insulin (V_30INS_ and V_100INS_) for 12 days increased the levels of the antioxidant GSH in the pancreatic tissue compared to the diabetic group (DM).

The weights of the liver, kidneys, and adrenal glands did not differ among the groups.

As expected, food intake and water consumption increased by approximately 30% and 140%, respectively, in the diabetic group compared with the control group (NG). This pattern was not altered in the presence of insulin or Vox2.

## 3. Discussion

Until recently, insulin was the only pharmacotherapeutic option for type 1 diabetes (T1D) [50]. In this context, vanadium has received attention as a potential adjuvant therapy for diabetes owing to its lack of deleterious effects on normal metabolism and homeostasis and because no toxicity has been reported [10,51]. Many vanadium compounds have been reported to become less toxic upon coordination with organic ligands [52,53,54].

We have recently reported that a water-soluble vanadium oxalate compound Vox2 at low doses (0.1 µM) improved the uptake of glucose analog in cells (HepG2) with insulin resistance similar to insulin (standard treatment) [48].

To examine the effects of Vox2 on behavior, we used the open-field test, a well-established paradigm to measure locomotion, depression-like states, and anxiety-like behaviors in animals [55]. As shown in Figure 2, there were no changes in the mean locomotor activity (Figure 2A), exploratory drive (Figure 2B), feed (Figure 2C), or kaolin intake (Figure 2D) attributable to Vox2 administration at all doses tested (30 and 100 mg/kg). Therefore, Vox2 did not have a sedative or stimulatory effect, nor did it cause drug-induced nausea.

Diabetes was successfully established as all six diabetic groups presented glucose concentrations above 250 mg/dL (Figure 3A). Acute administration (3 h) of Vox2 (Figure 3B) or prolonged administration (12 days) (Figure 3D) by oral gavage did not present an insulin-mimetic effect in diabetes-treated animals, as reported in other studies with vanadium complexes, such as bis-(maltolato)oxidoovanadium(IV) [25,56] (VO(malto)_2_), polyoxidometalates [57], and peroxidovanadium complexes [48,53]. However, there was a reduction in plasma glucose levels in the diabetic treated group to euglycemic levels (<9 mmol/L) after 4 weeks of treatment with the compound Bis(maltolato)oxidovanadium(IV) at 0.37 mmol/kg [25]. Intrajugular vein injections of potassium bisperoxido(1,10phenanthroline)oxidovanadate 0.6 µmol/kg [58] produced a decrease as marked as that observed following insulin administration.

In contrast, it has been demonstrated that vanadyl compounds [59] and oxidovanadium(IV)-malate complex [60] can enhance the effectiveness of administered insulin in animal models. Similarly, vanadyl sulfate improves hepatic and muscle insulin sensitivity in patients with type 2 diabetes [61].

Vox2 combined with insulin improved glycemic control by up to 2 times compared to standard insulin treatment in a dose-dependent manner (Figure 3C). Vox2 at 100 mg/kg (0.156 mmol/kg) in combination with insulin (V_100INS_) was effective in lowering plasma glucose levels to the point of stable euglycemia in diabetic animals within 2 days of treatment initiation (Figure 3C). In studies with similar experimental designs, the oral [V^IV^O(octd)] complex at 50 mg/kg/day for 21 days [62] produced a decrease that was as marked as that observed following insulin administration. We have previously evaluated the [VO(bpy)(mal)]·H_2_O insulin-like properties. Similarly, this compound was found to improve glycemia only in association with insulin; however, 30 mg/kg (0.0469 mmol/kg) of [VO(bpy)(mal)]·H_2_O, the maximum dose tested, associated with insulin decreased blood glucose concentrations by 30% compared to that in the insulin treatment [60]. Comparatively, Vox2 was more efficient, promoting and associated with insulin and a return to glycemia, similar to the control group at concentrations of 30 and 100 mg/kg.

Studies on other oxidovanadium(IV) compounds with different ligands that present exceptionally effective antidiabetic properties have been reported in the literature [11]. It is difficult to compare the different effects given the type and varying levels of glycemia and residual insulinemia in streptozotocin (STZ)-induced diabetic rats. Similarly, the vanadium complex decomposes after administration, and other complexes can form with cellular components [63]. Alternatively, the differences in potency between vanadium compounds could be related to their insulin-like properties. These differences in insulin-like properties are attributed to higher redox stability and higher hydrophilic stability [64]. The chemical species (forms) of vanadium plays a key role in the anti-diabetic action, although it is not clear if the chemical valence of V^V^ or the related coordinates are the most important for its anti-diabetic effect [65].

STZ-induced diabetes is characterized by a severe loss of body weight, hyperphagia, and polydipsia, which is consistent with the previous studies [52,66], and the alterations in total cholesterol, non-HDL, urea [67], and amylase [68] observed in this study (Table 1) were the result of diabetic induction. In this context, an increase in plasma total cholesterol is related to insulin deficiency because of its high absorption [69,70].

STZ-induced diabetes is associated with impairment of the amylase-release mechanism and/or its synthesis, and insulin treatment can reverse pancreatic insufficiency in diabetic animals [71,72]. Vox2 associated with insulin (V_30INS_ and V_100INS_) promoted the restoration of amylase activity compared to that in the diabetic group, suggesting a protective effect on the exocrine pancreas.

An increase in urea concentration in diabetic animals was expected, resulting in the reabsorption of water and urea together without necessarily demonstrating kidney damage, which can also be inferred from the maintenance of creatine concentration without change in the groups under study. The lack of urea restoration after treatment has been reported in other studies [67], and treatment with insulin or Vox2 associated with insulin (V_30INS_ and V_100INS_) in diabetic rats decreased food and water intake compared to the DM group.

Although it has been reported that vanadium complexes coordinated with organic ligands are less toxic [52,53,54], they can be converted to simple vanadate salts and/or oligomers in the biological system, and vanadate toxicity must be considered [73]. In vitro studies have shown that vanadium acts as a phosphate analog and, as such, interferes with various ATPases, phosphatases, and phosphate-transfer enzymes. The effect of vanadium on various enzymes may be responsible for the diverse effects observed in animals exposed to vanadium. However, little information is available regarding the mechanism of vanadium toxicity in vivo [74]. The toxicity of Vox2 in diabetic animals has been examined using weight loss, need for rehydration, and elevated serum parameters such as alkaline phosphatase (ALP) and aspartate aminotransferase (AST), which are signs of liver dysfunction and survival in various studies [75,76]. In relation to ALP, the only group that showed a reduction in enzymatic activity was V_100INS_. Different vanadium compounds have shown inhibition of ALP [77]. New studies with Vox2 are needed to confirm this effect, observed only in association with insulin in this study. Animals treated with Vox2 alone did not show any differences in the parameters observed in diabetic animals (Table 1). Therefore, Vox2 does not cause side effects such as gastrointestinal discomfort, diarrhea, or hepatic or renal toxicity.

Dyslipidemia is a major complication of diabetes [78]. Some studies have reported that vanadium exerts antilipidemic effects in animal models [67,79] and patients with type 2 diabetes (T2D) [61].

However, treatment with Vox2 for 12 days did not reduce the lipid profile of diabetic rats in this study (Table 1). The expected improvement of the lipid profile is attributed to the enhanced glucose utilization [80], increased phospholipase A2 activity [81], correction of lipogenic enzymes such as G6PD [82], and inhibition of 3-hydroxy-3-methylglutaryl co A [83], which effects insulin mimetics. As Vox2 did not alter glucose uptake measured by glycemic control (Figure 3B–D), this may be an explanation for the lack of effect on the lipid profile observed. Nevertheless, it was reported that some compounds showing a tendency to lower diabetic hyperglycemia did not lower diabetic hyperlipidemia, and vice versa [63]. Therefore, Vox2 does not affect cholesterol levels, as reported for other oxidovanadium compounds [84,85], and the period in which the experiments were conducted in this study was insufficient to detect an anti-lipemic effect. It has been reported that the metabolic effects of vanadium are dose-dependent and require more than 4 weeks for a complete response [84,85,86,87].

Hyperglycemia is a cause of oxidative stress conditions [88]. Diabetes affects antioxidant enzyme activity, which further increases oxidative stress [89]. As we found that Vox2 has antioxidant effects in vitro, as reflected by the DPPH free radical scavenging activity (Figure S4A) and FRAP (Figure S4B), we investigated the liver and pancreatic tissues. We did not identify changes in the markers of oxidative stress (GSH and LPO) in the liver in our study (Table 1). However, Vox2 associated with insulin increased the GSH levels in the pancreatic tissue after 12 days of treatment, improving the antioxidant capacity of these tissues compared to the diabetic group (Table 1). It has been reported that the level of intracellular NADPH declined during insulin deficiency because of defective glucose oxidation; thereby, the level of GSH decreased [89]. In agreement with our results, the previous studies have indicated that vanadium compound is capable of restoring the activities of antioxidant enzymes to normal levels in different tissues of STZ-induced diabetic rats [29,90,91]. Similarly, Trevino et al. (2016) reported that metforminium decavanadate (MetfDeca) protects pancreatic beta cells in DM1 rats, suggesting the possible regeneration of these cells by recovering their insulin levels [92]. The reduction in GSH was observed in different models of diabetes and associated with the duration of diabetes and the severity of hyperglycemia [93,94], and it has been postulated that insulin signaling regulates myocardial GSH through a coordinated activation of pathways involved in GSH synthesis and NADPH production [95]. Vox2, in the presence of insulin (V_30INS_ and V_100INS_), promoted an increase in pancreatic GSH of more than 50% compared to the NG group. This finding, which requires further studies for confirmation, may suggest a synergism of Vox2 and insulin on GSH activity.

Oxidative stress in diabetes in humans plays roles in both the origin of the disease and in increasing secondary complications [88], resulting in the production of free radicals, especially in the pancreas, which is a major cause of insulin resistance in both type 1 [96] and type 2 diabetes [97]. Associated with the fact that the type 1 diabetes treatment regimen is associated with weight gain [98], and a higher BMI is related to worse metabolic control [99,100], there is a need for adjuvant therapy with insulin, which improves glycemic control and reduces insulin requirements [101].

Taken together, oral treatment with Vox2 in STZ-induced diabetic rats improved glycemic control and the activities of pancreatic enzymatic antioxidants when associated with insulin therapy. These results pointed to Vox2 as a potential oral adjuvant pharmacotherapy for insulin in the treatment of type 1 diabetes, promoting improvement in glycemic control associated with a reduction in insulin dose and pancreatic antioxidant status.

## 4. Materials and Methods

### 4.1. Synthesis of the Oxidovanadium(IV) Complex—Vox2

The synthesis of Vox2 was carried out using type III deionized water with a resistivity of 0.2857 MΩ·cm purified in a DE1800 Evolution Deionizer. Vanadium pentoxide (V_2_O_5_, 99.6%), triethylamine (Et_3_N, 99%), 1,3-butanediol (1,3-bd, >98%), and oxalic acid dihydrate (H_2_C_2_O_4_·2H_2_O, 99.5%) all from Sigma-Aldrich (São Paulo, Brazil) were used as received.

Carbon, hydrogen, and nitrogen contents were determined by combustion using a Perkin Elmer 2400 Series II Elemental Analyzer (Waltham, MA, EUA). Powder X-ray diffraction of Vox2 was evaluated by the Shimadzu diffractometer XRD600 (Barueri, SP, Brazil) equipped with a Cu-target tube (Cu-Kα, λ = 1.5418 Å) with a 2θ range of 5–50°. The calculated diffractogram was generated from a single-crystal crystallographic information (CIF) file using Mercury 4.0 software [102]. Infrared (IR) data were recorded from KBr pellets on a Bruker VERTEX 70v spectrophotometer (Bruker Madison, WI, USA) with a resolution of 4 cm^−1^ in the 400–4000 cm^−1^ range. X-band EPR spectra (9.5 GHz) were recorded at 77 K from aqueous solutions using a Bruker EMX-Micro spectrometer.

Preparation of the oxidovanadium(IV) complex: The (Et_3_NH)_2_[{VO(OH}_2_)(ox)_2_(µ–ox)], designated as Vox2, was synthesized by adapting the procedure described by Baptistella et al. [48]. Briefly, 8.300 g of H_2_C_2_O_4_·2 H_2_O (66.0 mmol) was added to a dark yellow dispersion of V_2_O_5_ (4.000 g, 22.0 mmol) in 80.0 mL of water. The reaction mixture was stirred at 60 ℃ for 2 h, producing a dark blue solution, to which 2.00 mL of 1,3-butanediol (22.0 mmol) and 6.10 mL (44.0 mmol) of triethylamine were added. The resulting dark green solution was filtered and poured into a beaker after 2 h. Light greenish-blue crystals formed after 72 h; the supernatant was removed, washed with ethyl alcohol (3 × 5.0 mL), and finally, dried in air to obtain 8.450 g of Vox2; a yield: of 64%, based on vanadium. The product was highly water-soluble. Elemental analysis calculated (%) for the compound C_18_H_36_O_16_N_2_V_2_ (638.37 g mol^−1^) was C, 33.86; H, 5.68; N, 4.38; and it is found: C, 33.97; H, 5.76; N, 4.32%. IR (KBr, cm^−1^, s = strong, m = medium, w = weak, br = broad): 3415(b) [ν(O–H), ν(N–H)], 1691(m) and 1637(m) [ν(C–O)], 1400(s) [ν(CO)], 1263(s) [ν(CO_2_)], 1161(w) [ν(C–H)], 989(s) [ν(V=O)], 811(s) [ν(V–O)], 549(m) and 489(m) [δ(V-O)].

Crystal data for Vox2: One light greenish-blue crystal of complex Vox2 was analyzed on a Bruker D8 Venture diffractometer with a Photon 100 CMOS Bruker Madison, WI, USA (detector using Mo-Kα radiation (0.71073 Å) at 300 K. The structure of (Et_3_NH)_2_[{VO(OH}_2_)(ox)_2_(µ–ox)] was confirmed via single-crystal X-ray diffraction analysis. Monoclinic, space group P21/n (no. 14), a = 7.7183(14) Å, b = 18.529(3) Å, c = 9.6202(16) Å, β = 97.047(6), V = 1365.4(4) Å^3^. Z = 2, Dc = 1.553 mg m^−3^, F(000) = 664.

### 4.2. Protocols Followed for In Vivo Studies with Vox2 Complex on Wistar Rats

This research was approved by the Ethics Committee on Animal Use (CEUA/BIO-UFPR 1381) and followed the international rules for laboratory animal welfare. The animals were provided by the vivarium of UFPR.

Vox2 solutions: The light greenish-blue crystals of complex Vox2 are very soluble in water (100 mg/mL) producing blue solutions (Figure S7), for which the pH measured varied from 4.6 at 1.0 mmol/L to 5.4 at 0.1 mmol/L. The solutions used in the biological studies were prepared daily in ultra-pure water in the desired concentrations prior to use. The concentrations were calculated in mg/kg based on the weight of each animal (average of 200 g) to a final volume of 1.0 mL.

Animals: 70 male Wistar rats (*Rattus norvegicus*), 200–250 g were used to evaluate the vanadium compound effects in behavior (Open-Field Test) and in diabetes (Animal Trials with Vox2) experiments. The animals were group housed, four per cage (41 × 32 × 16.5 cm), at room temperature (21 ± 2 °C), and under a 12 L:12 D cycle (lights on at 7:00–19:00 h). Standard pellet food (Altromin 1326, Altromin, Lage, Germany) and water were provided ad libitum. The forage in the cages was replaced daily.

#### 4.2.1. Monitoring and Analysis of Behavior

##### Open-Field Test

The 22 animals were randomly divided into 3 groups. Separate rats (n = 7 or 8) were used for each dose and the animals were used once. The naïve control group (n = 7) received the vehicle only at the same volume as the test animals: Naïve V_30_ (n = 7) and Naïve V_100_ (n = 8).

The powdered compound Vox2 was dissolved in distilled water stirred at ambient temperature until a visually homogeneous mixture was achieved. All doses (1 mL) were administered volumetrically. Each animal was dosed by oral gavage after 12 h of fasting using a 7 cm gavage needle. Doses of 30 and 100 mg/kg were soluble in up to 1.0 mL of the solution. Higher doses, such as 300 mg/kg, precipitated and prevented application at full concentrations.

The behavioral experiments were performed in an observation chamber between 09:00 and 11:00 am using a black wooden open-field apparatus (50 × 40 × 63 cm). Locomotor activity was assessed simultaneously at 1-min intervals by interrupting 10 equally spaced infrared light beams in the open field. The animals were transferred to observation chambers (30 × 20 × 13 cm) containing standard food pellets (Altromin 1326 Altromin, Lage, Germany), kaolin, and water 1 h before testing. Kaolin was used to evaluate the emetic response in rats. Rats cannot vomit but show pica behavior, that is, eating non-nutritive substances such as kaolin [103]. Following this period, the animals were placed individually in one corner of an unfamiliar open field facing the center. Each rat was observed from the outside by remote monitoring for 5 min and the number of crossed quadrants was recorded. The open field was cleaned after each animal using 30% ethanol. To exclude batch and seasonal variations, control groups (n = 7) treated with the vehicle were tested at irregular intervals between the vanadium-treated groups.

Locomotor and exploratory activities were measured by counting the number of quadrants and the crossed central quadrants, respectively. Autonomic nervous system stimulation and state of anxiety in these animals were evaluated by counting the foci of urine or fecal material left by the animals during their time in an open field [104,105].

#### 4.2.2. Vanadium Compound (Vox2) Effect in Diabetes

The 48 animals were randomly divided into 7 groups. These were Group NG (n = 10): normoglycemic group, the negative control, non-diabetic, normal healthy rats treated with intraperitoneal citrate buffer (10.0 mmol/L, pH 4.5); Group DM (n = 8): diabetes mellitus group, the positive control, induced diabetic rats untreated; Group INS (n = 6): induced diabetic rats treated with insulin, the control treated; Group V_30_ (n = 6): induced diabetic rats treated with 30 mg/kg of Vox2 compound; Group V_100_ (n = 6): induced diabetic rats treated with 100 mg/kg of Vox2 compound; Group V_30INS_ (n = 6): induced diabetic rats treated with insulin plus 30 mg/kg of Vox2 compound; and Group V_100INS_ (n = 6): induced diabetic rats treated with insulin plus 100 mg/kg of Vox2 compound. Vox2 was administered via gavage, once a day (30 mg/kg and 100 mg/kg at 9:00 a.m.). Insulin treatment consisted of daily Caninsulin MSD Rahway, NJ, USA (10 mL, 40 IU/mL) subcutaneous injections (2 IU (International Unit) at 9:00 am and 4 IU at 5:00 p.m.). Blood glucose levels were measured every two days during the 12-day experiment. The amount of food and water consumed was determined daily and compared to that of the controls. Figure S5 summarizes the protocol used to determine the effect of the vanadium compound (Vox2) in the diabetes experiment.

Induction of Diabetes: The induction was performed after a 12-h fast with a single intraperitoneal injection of 60 mg/kg streptozotocin (STZ) in citrate buffer (10 mM, pH 4.5) [106]. The diabetes state was confirmed three days after the STZ injection using a blood glucometer test (AccuCheck active, Roche Diagnostic, Burgess Hill, UK), and rats with a blood glucose level ≥250 mg/dL were diagnosed as diabetic [107].

Selection of Vox2 dose: The Vox2 compound was initially analyzed using a neurobehavioral test to determine the doses to be used in the prolonged stage of this study. The doses of 30 and 100 mg/kg were chosen based on range extracted from the literature [14,60,62] available and on solubilization in distilled water. The oral doses tested did not affect animals neurobehavior. Higher doses, such as 300 mg/kg, precipitated and prevented application at full concentration.

Euthanasia and sample collection: At the end of the experiment, animals were anesthetized with thiopental anesthesia (100 mg/kg, intraperitoneal) and submitted to decapitation.

Whole blood was collected in a tube with EDTA (2 mL; BD Vacutainer K_2_EDTA tubes) and in tubes with heparin (8 mL, BD Vacutainer Sodium heparin tubes, Franklin Lakes, NJ, EUA), which were subsequently centrifuged (less than 10 min) to obtain plasma, and both were stored at −80 °C to evaluate biochemical parameters. Liver, kidney, and adrenal tissues were collected for oxidative stress and histological evaluation.

### 4.3. Organ Weights and Histopathology

The livers, adrenal glands, and kidneys, important organs for the metabolism and diabetes, were weighed. The fixed tissues were trimmed, processed, embedded in paraffin, sectioned using a microtome, placed on glass microscope slides, stained with hematoxylin and eosin, and examined by light microscopy using an Axio Imager Z2 epifluorescence microscope (Carl Zeiss, Jena, Germany) equipped with an automated scanner (MetaViewer version 2.0.100; MetaSystems, Altlussheim, Germany). The slides were prepared at the Technical Center for Histopathology of Curitiba by a certified veterinary pathologist.

The pathological analysis of fixed liver tissues was evaluated for the presence of hepatocyte congestion, inflammatory infiltration, fibrosis, and vacuolization. Hepatic injuries were scored as follows according to the semi-quantitative scoring system [108]: grade 0, within normal limits; grade 1, minimal; grade 2, mild; and grade 3, moderately marked.

### 4.4. Oxidative Stress Parameters

In vitro determination of Vox2 free radical scavenging activity: The scavenging activity of different concentrations of Vox2 (1, 3, 10, 30, 100, 300, and 1000 μg/mL) was determined using two methods: DPPH (2,2 diphenyl-1-picrylhydrazyl free radical), using an adapted method from Chen et al. (2004) [109]; and ferric reducing ability of plasma (FRAP) using the antioxidant trolox, through an adaptation of the method proposed by Urrea-Victoria et al. (2016) [110]. Both methods were performed in 96-well microplates, and the absorbance was read at 517 nm and 595 nm using a microplate reader (Synergy HT, Biotek, VT, USA). Ascorbic acid (50 μg/mL) and distilled water were used as positive and negative controls, respectively.

In vivo Vox2 effect in redox status: The fragment tissues of the liver and pancreas were homogenized in potassium phosphate buffer (pH 6.5, 1:10) and centrifuged at 10,000× *g* at 4 °C for 20 min. The homogenates were used to measure reduced glutathione (GSH) and lipid peroxidation (LPO), while the supernatant was used to measure total tissue protein levels.

The contents of LPO were determined using a method based on the rapid peroxide-mediated oxidation of Fe^2+^ to Fe^3+^ under acidic conditions, which forms a Fe^3+^-xylenol orange complex in the presence of xylenol orange [111].

The GSH content was estimated using a modified Ellman’s method [112] based on the development of a stable yellow complex with 5,5-dithio-bis-(2-nitrobenzoic acid, DTNB, Ellman’s reagent).

The protein content of the tissue homogenate was estimated using the Bradford method [113]. All of the tests were performed in 96-well microplates, and the absorbances were read in a microplate reader (Synergy HT, Biotek, VT, USA).

### 4.5. General Clinical Observations and Biochemical Markers

Body weight, diet, and drinking water were monitored daily throughout the experiment. The blood glucose levels in each animal were checked every two days for 12 days. Blood samples were obtained by pricking the tail, and blood glucose levels were determined using an Accu-Chek Active (Roche Diagnostic, Burgess Hill, UK) blood glucose monitor. On the first day (day 5), i.e., when the treatment was initiated, glycemia was monitored at 30, 60, 120, and 180 min after the drugs administration (Figure S5).

Plasmatic biochemical levels, including alanine aminotransferase (ALT), aspartate aminotransferase (AST), alkaline phosphatase (ALP), lactate dehydrogenase (LDH), amylase, total cholesterol, high-density lipoprotein cholesterol (HDL-C), triglycerides, urea, creatinine, uric acid, total protein, albumin, and glucose, were measured at the end of the experiment (12 day of treatments) and quantified using an automated clinical chemistry analyzer (Labmax 400; Labtest Diagnostics, Lagoa Santa, MG, Brazil) with reagents, standards, and calibrators provided by the equipment manufacturer. The non-HDL-C levels were also calculated (non-HDL-C = total cholesterol-HDL-C).

### 4.6. Statistical Analysis

All experimental values were reported as mean ± SD. Normality was tested using the Kolmogorov–Smirnov test. The statistical significance of the differences was determined using one-way ANOVA, followed by Tukey’s honest significant difference test. Statistical significance (*p*) < 0.05 was considered significant.

All statistical analyses were performed using Statistica for Windows 10.0 software (TIBCO Software Inc., 2020, Palo Alto, CA, EUA) and GraphPad Prism version 10.0.0 for Windows, GraphPad Software, Boston, MA, USA, www.graphpad.com (accessed on 27 February 2024).

## 5. Conclusions

The studied complex oxidovanadium(IV), (Et_3_NH)_2_[{VO(OH}_2_)(ox)_2_(µ–ox)] or Vox2, with a water-soluble oxalate species, used together with insulin at concentrations of 30 and 100 mg/kg, promotes a significant improvement in blood glucose levels in STZ-diabetic rats without relevant toxicity indicators in a 12-day experiment. Vox2 demonstrated potential for its add-on therapeutic activity as a promising drug for new studies to compose the treatment of diabetes.

## Figures and Tables

**Table 1:** Nutritional, serum biomarkers and rat organ statuses of the study groups.

Parameters	NG	DM	V_30_	V_100_	INS	V_30INS_	V_100INS_	*p*
Lipidic profile								
Total Cholesterol, mg/dL	41.7 ± 9.8	**65.7** ** ± 8.3 ^a^**	**71.0** ** ± 30.8 ^a^**	**61.8** ** ± 9.1 ^a^**	52.6 ± 9.6	50.7 ± 9.3	58.8 ± 10.2	**<0.001**
Triglycerides, mg/dL	99.5 ± 39.7	188.8 ± 99.7	99.0 ± 46.2	84.0 ± 17.2	133.0 ± 60.4	155.3 ± 97.1	123.6 ± 66.9	0.118
HDL-C, mg/dL	34.0 ± 6.6	**51.1** ** ± 10.3 ^a^**	46.5 ± 9.4	**47.5** ** ± 6.8 ^a^**	42.4 ± 8.6	37.8 ± 4.0	41.4 ± 7.3	**0.005**
nHDL, mg/dL	7.7 ± 3.2	**15.4** ** ± 4.6 ^a^**	13.4 ± 3.3	14.3 ± 6.8	10.2 ± 3.1	11.2 ± 3.7	**17.4** ** ± 5.6 ^a^**	**0.010**
Kidney function								
Urea, mg/dL	35.4 ± 3.9	**53.3** ** ± 6.8 ^a^**	**51.5** ** ± 7.7 ^a^**	**50.8** ** ± 4.6 ^a^**	**56.7** ** ± ** **8.6 ^a^**	**57.4** ** ± 4.7 ^a^**	47.4 ± 13.3	**<0.001**
Creatinine, mg/dL	0.12 ± 0.04	0.07 ± 0.04	0.09 ± 0.10	0.11 ± 0.07	0.14 ± 0.04	0.06 ± 0.04	0.13 ± 0.04	0.237
Uric acid, mg/dL	0.4 ± 0.3	0.3 ± 0.2	0.8 ± 1.0	0.8 ± 0.6	0.8 ± 0.4	0.7 ± 1.0	0.6 ± 0.2	0.790
Nutritional profile								
Total Protein, g/dL	6.2 ± 0.6	6.1 ± 0.5	6.1 ± 0.8	5.6 ± 0.7	6.2 ± 1.0	5.8 ± 0.6	6.2 ± 0.6	0.703
Albumin, g/dL	2.8 ± 0.2	2.7 ± 0.2	2.6 ± 0.1	2.4 ± 0.4	2.8 ± 0.4	2.6 ± 0.3	2.7 ± 0.2	0.388
Liver function								
AST, U/L	150 ± 91	165 ± 66	127 ± 63	175 ± 75	191 ± 74	200 ± 128	200 ± 58	0.626
ALT, U/L	71 ± 15	74 ± 30	78 ± 33	90 ± 12	82 ± 13	82 ± 23	85 ± 7	0.675
ALP, U/L	265 ± 62	465 ± 29	440 ± 111	260 ± 60	472 ± 189	445 ± 273	**151** ** ± 60 ^b^**	**<0.001**
LDH, U/L	1449 ± 677	1607 ± 619	1131 ± 850	1654 ± 481	1634 ± 640	1496 ± 686	1755 ± 593	0.687
Pancreatic function								
Amylase, U/L	478 ± 44	**277** ** ± 50 ^a^**	**239** ** ± 92 ^a^**	**299** ** ± 62 ^a^**	389 ± 102	400 ± 127	**452** ** ± 101 ^b^**	**<0.001**
Oxidative stress Liver								
GSH, µg/g tissue	1628 ± 750	1755 ± 1098	1834 ± 1030	1927 ± 1196	1808 ± 954	2533 ± 1446	1609 ± 1139	0.726
LPO, nmol/mg protein	8.64 ± 2.4	10.5 ± 5.9	12.71 ± 8.5	6.76 ± 1.4	8.05 ± 0.9	8.72 ± 5.0	8.95 ± 1.8	0.345
Tissue protein, mg/mL	12.8 ± 2.8	13.8 ± 1.7	12.2 ± 3.4	10.7 ± 2.8	12.9 ± 1.9	11.8 ± 2.1	12.3 ± 1.4	0.319
Pancreas								
GSH, µg/g tissue	841 ± 290	557 ± 433	328 ± 159	505 ± 269	683 ± 344	**1417** ** ± 551 ^b^**	**1382** ** ± 658 ^b^**	**<0.001**
Organ weights								
Liver, g	3.50 ± 0.3	3.79 ± 0.9	3.11 ± 0.6	3.33 ± 0.4	3.60 ± 1.0	3.60 ± 0.8	3.17 ± 1.1	0.643
Kidneys, g	1.25 ± 0.2	1.35 ± 0.2	1.39 ± 0.2	1.16 ± 0.1	1.28 ± 0.1	1.34 ± 0.2	1.18 ± 0.2	0.523
Adrenals, g	0.10 ± 0.05	0.06 ± 0.02	0.07 ± 0.06	0.16 ± 0.11	0.08 ± 0.06	0.06 ± 0.04	0.09 ± 0.05	0.051
Nutritional parameters								
Food intake (g/day)	26.0 ± 2.7	**33.9** ** ± ** **4.4 ^a^**	**34.4** ** ± ** **5.4 ^a^**	**31.7** ** ± ** **3.1 ^a,b^**	**30.8** ** ± ** **3.4 ^a,b^**	**31.1** ** ± ** **3.4 ^a,b^**	**30.8** ** ± ** **3.8 ^a,b^**	**<0.001**
Water (mL/day)	49.8 ± 5.4	**119.6** ** ± ** **9.5 ^a^**	**120.3** ** ± ** **9.6 ^a^**	**118.7** ** ± ** **9.1 ^a^**	**103.9** ** ± 8.3 ^a,b^**	**109.0** ** ± ** **8.3 ^a,b^**	**108.3** ** ± ** **9.1 ^a,b^**	**<0.001**
**Animals**								
weight (g)	284 ± 18	249 ± 28	**238 ± 32 ^a^**	**234 ± 21 ^a^**	247 ± 24	**238 ± 20 ^a^**	247 ± 29	**0.002**

Values are mean ± SD. Probability (p) one-way ANOVA. Significative comparison (*p* < 0.05) with NG group (control) “^a^” and with DM group (diabetic) “^b^” using Tukey correction. Significative differences are marked in bold. nHDL; non-HDL cholesterol (total cholesterol—HDL-cholesterol).

## Data Availability

The raw data supporting the conclusions of this article will be made available by the authors on request.

## Supplementary Materials

The following supporting information can be downloaded at: https://www.mdpi.com/article/10.3390/ph17040486/s1, Figure S1: A timeline of events relevant to vanadium use in diabetes; Figure S2: Powder X-ray diffraction patterns recorded (black line) and calculated (red line) for (Et_3_NH)_2_[{VO(OH}_2_)(ox)_2_(µ–ox)] (Vox2); Figure S3: X-band electron paramagnetic resonance (EPR) spectra registered for Vox2; Figure S4: In vitro scavenging activity of different concentrations of Vox2. (A) DPPH radical scavenging activity; Figure S5: Experimental design over 16 days with time points (D1–D16); Figure S6: Histology of hepatic tissue stained using hematoxylin and eosin; Figure S7: Freshly prepared aqueous solution of Vox2 in concentrations ranging from 10.0 to 0.1 mmol/L with the respective pH.
