# Supplementary figures and images for: A Non-Toxic Binuclear Vanadium(IV) Complex as Insulin Adjuvant Improves the Glycemic Control in Streptozotocin-Induced Diabetic Rats
Source: Pharmaceuticals (Basel). 2024 Apr 11;17(4):486. doi: 10.3390/ph17040486 (PMC11054326; doi:10.3390/ph17040486)

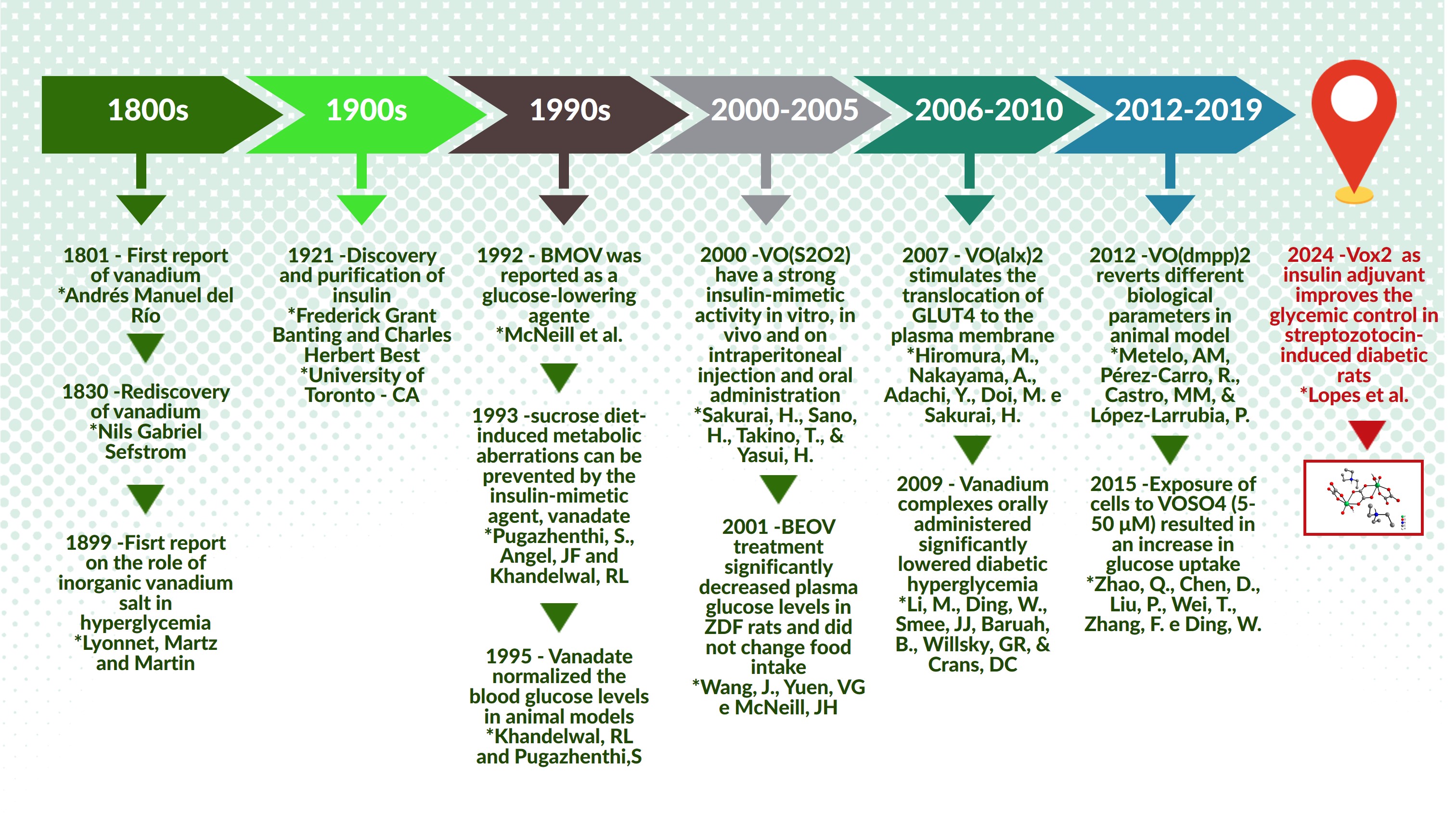

Supplement: Supplementary file 1 [file pharmaceuticals-17-00486-s001.zip › Figure S1. Timeline.jpg]

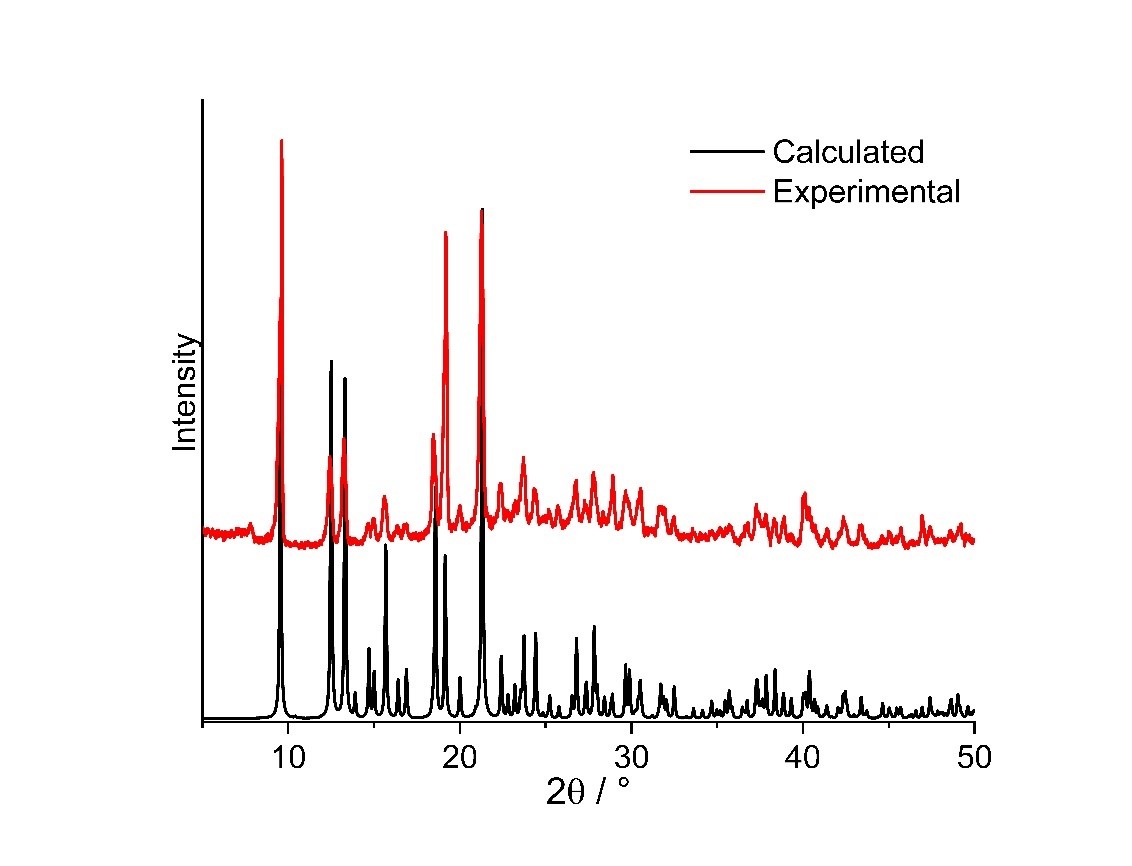

Supplement: Supplementary file 1 [file pharmaceuticals-17-00486-s001.zip › Figure S2. Powder X-ray.jpg]

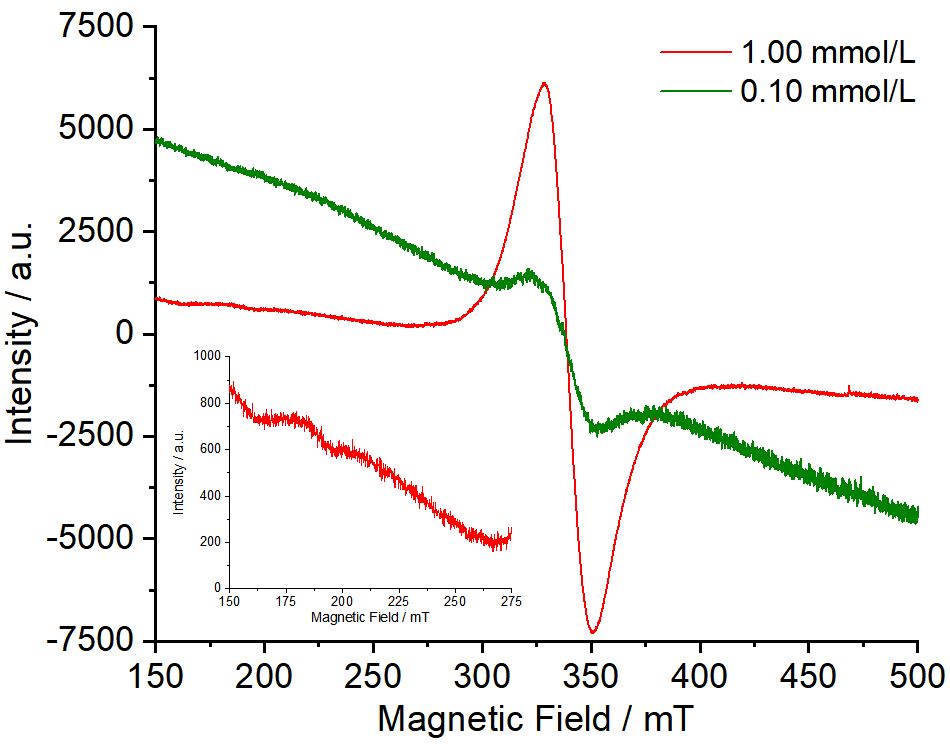

Supplement: Supplementary file 1 [file pharmaceuticals-17-00486-s001.zip › Figure S3. X-band EPR.JPG]

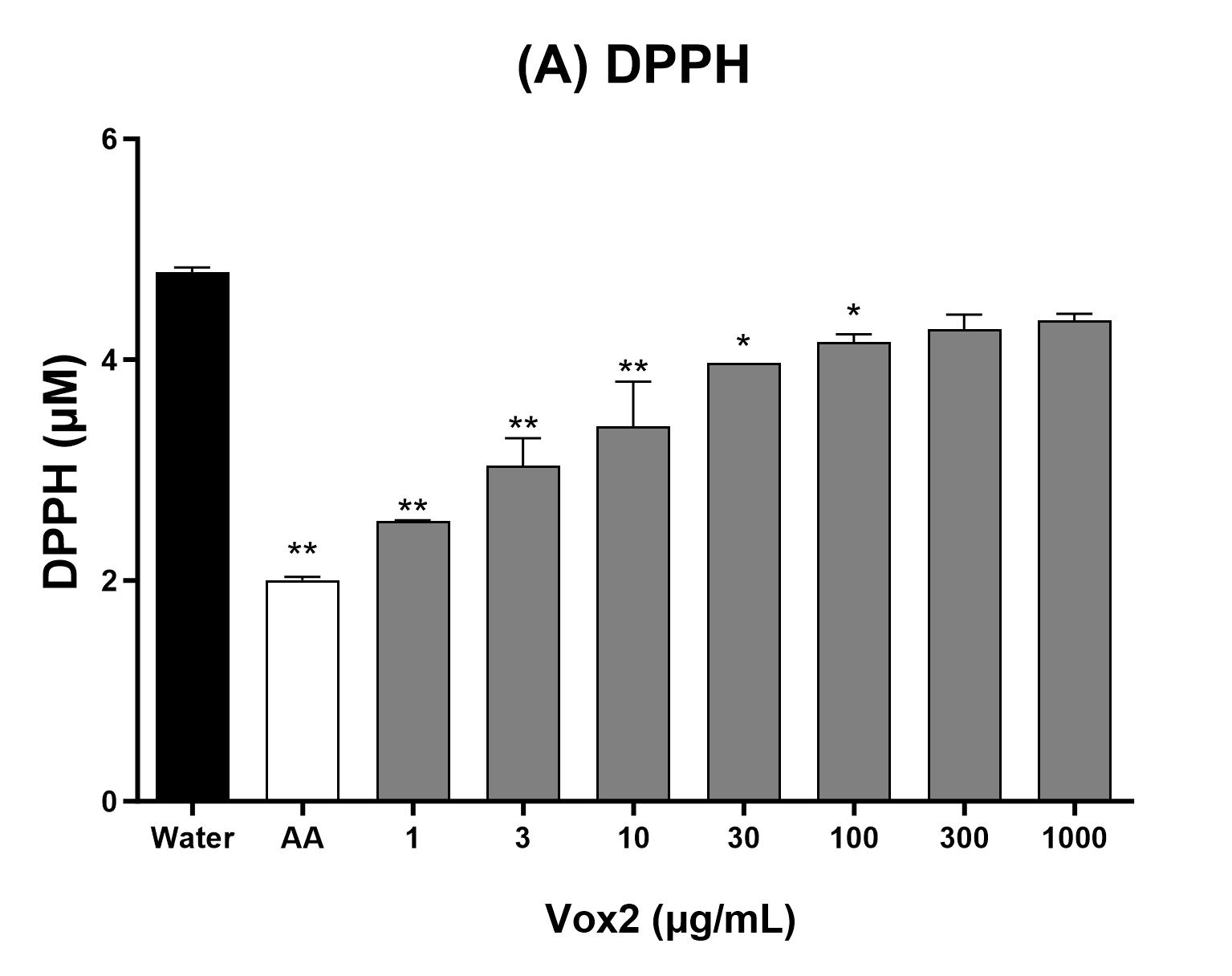

Supplement: Supplementary file 1 [file pharmaceuticals-17-00486-s001.zip › Figure S4 (A). DPPH.jpg]

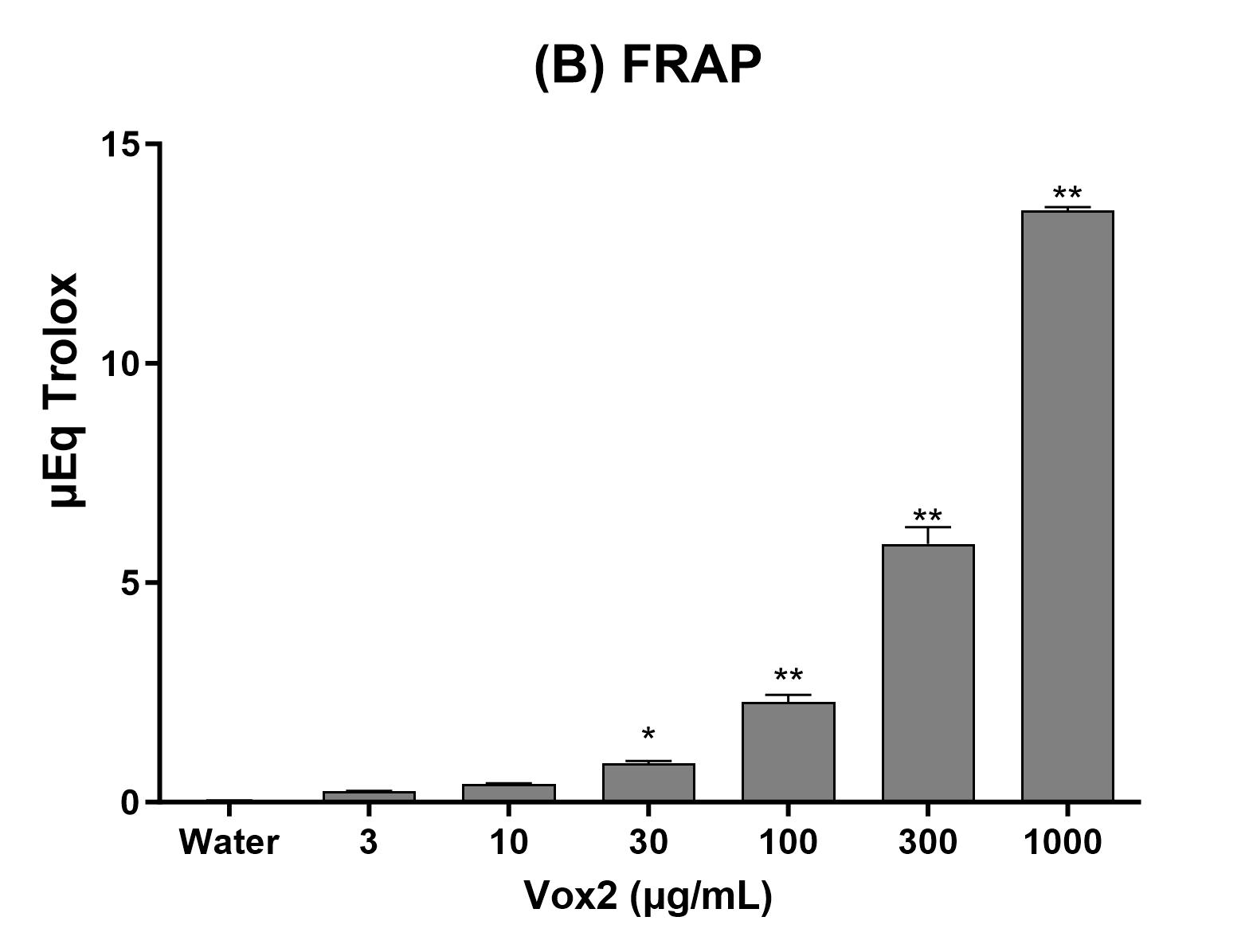

Supplement: Supplementary file 1 [file pharmaceuticals-17-00486-s001.zip › Figure S4 (B). FRAP.jpg]

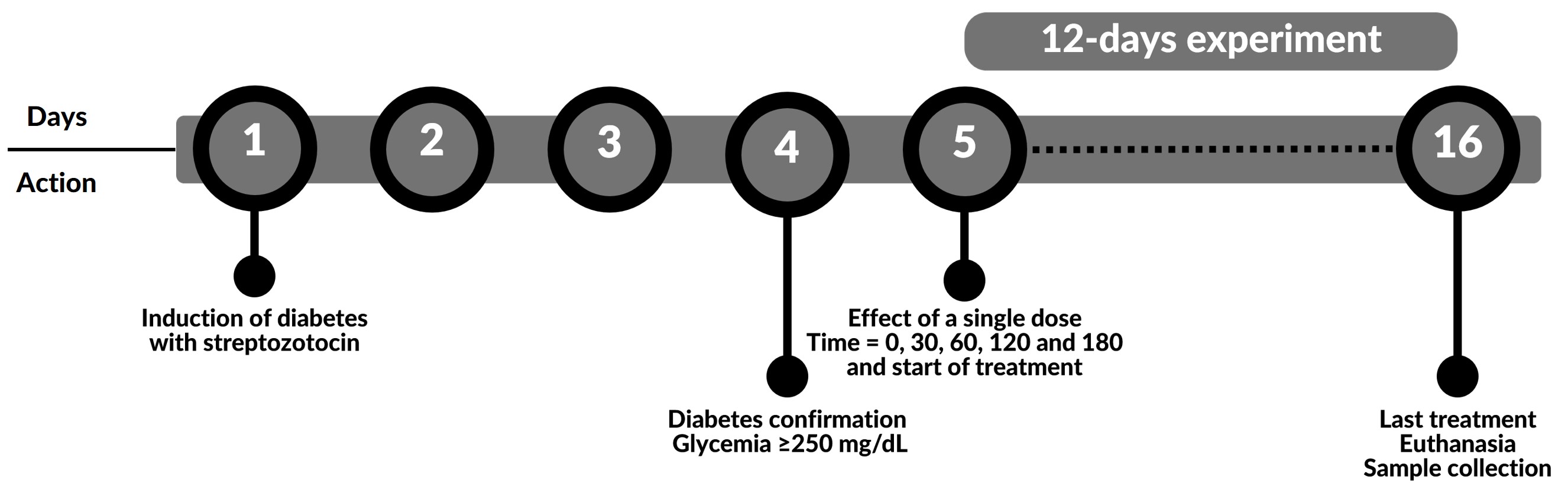

Supplement: Supplementary file 1 [file pharmaceuticals-17-00486-s001.zip › Figure S5. Experimental design.jpg]

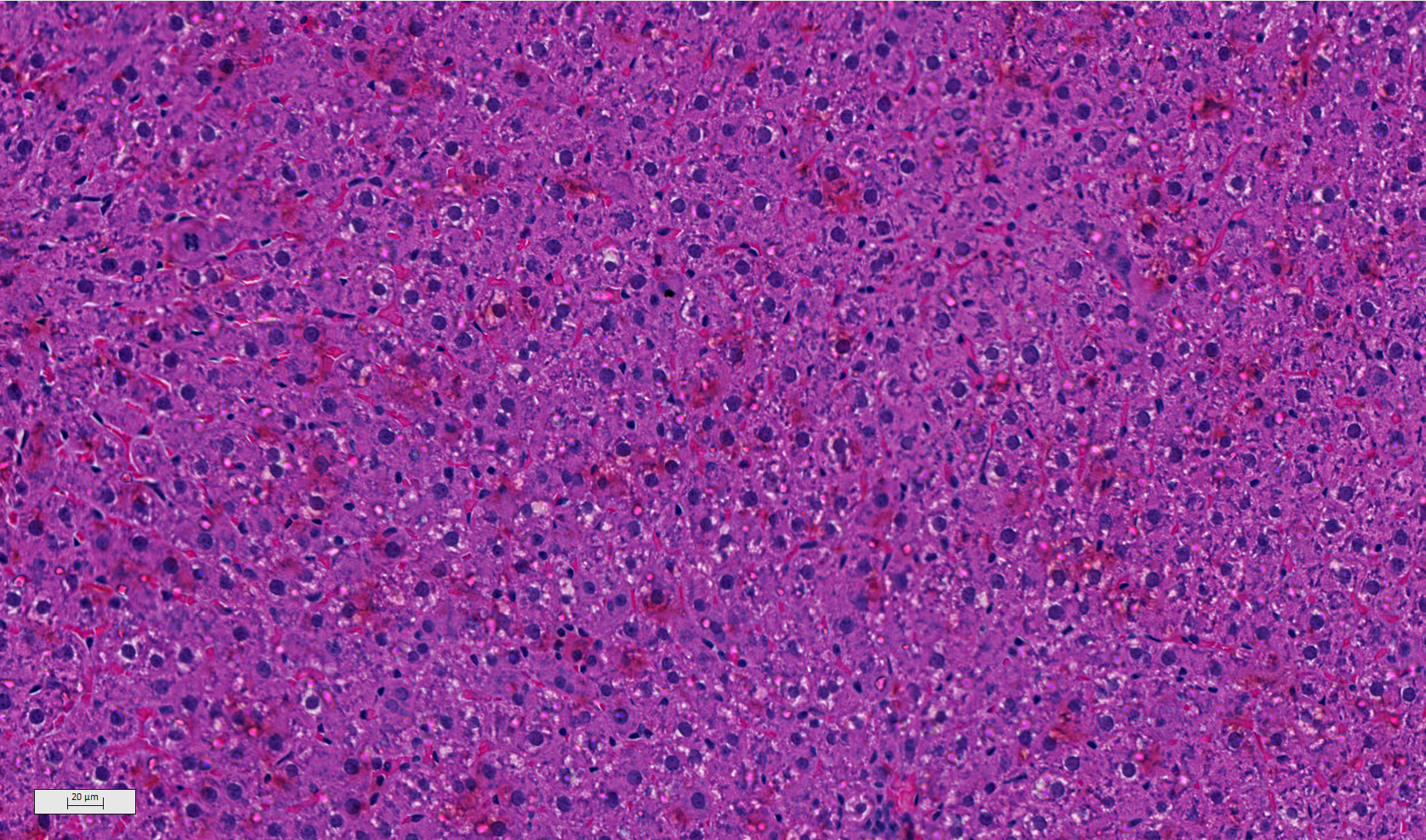

Supplement: Supplementary file 1 [file pharmaceuticals-17-00486-s001.zip › Figure S6 (A). NG.tif]

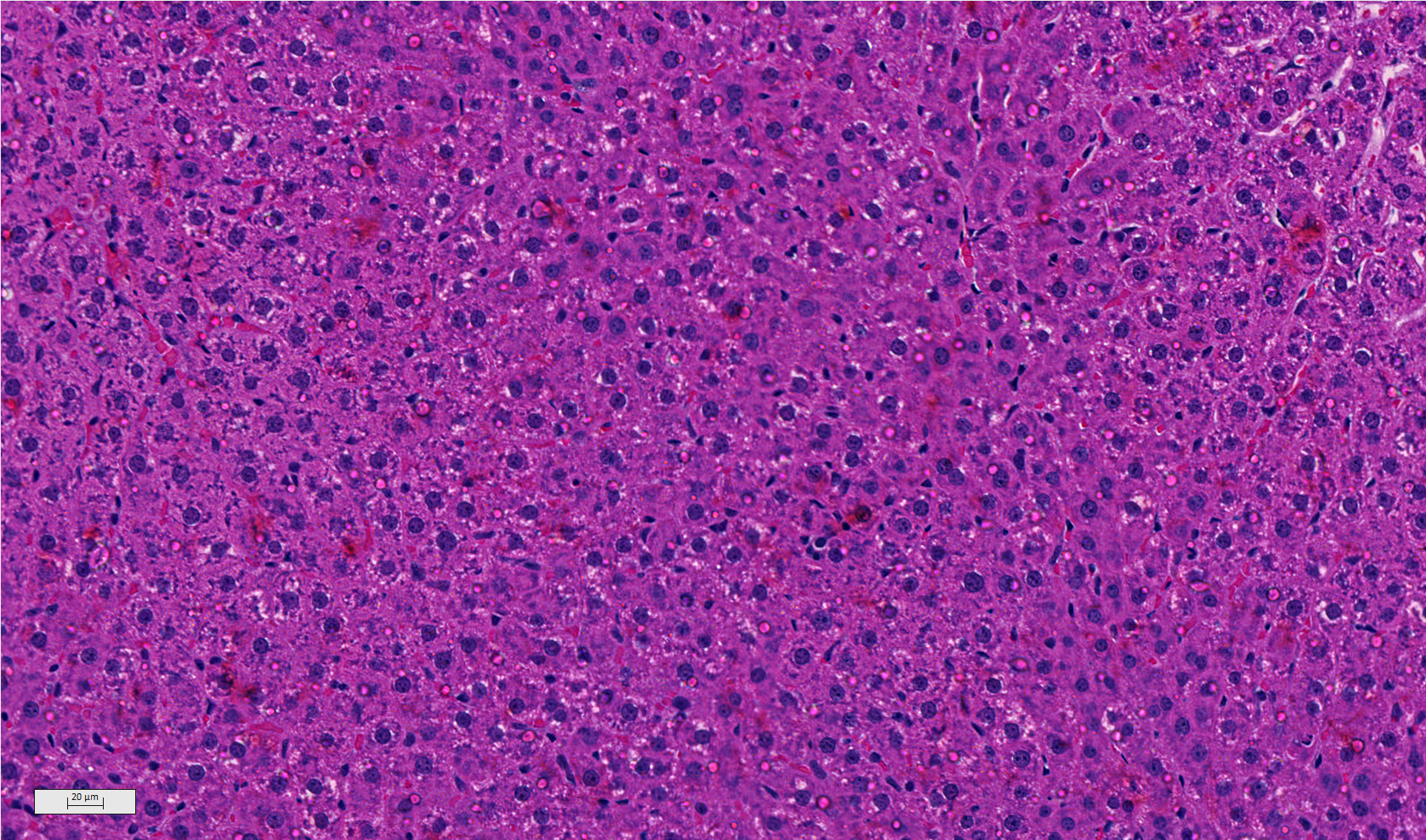

Supplement: Supplementary file 1 [file pharmaceuticals-17-00486-s001.zip › Figure S6 (B). DM.tif]

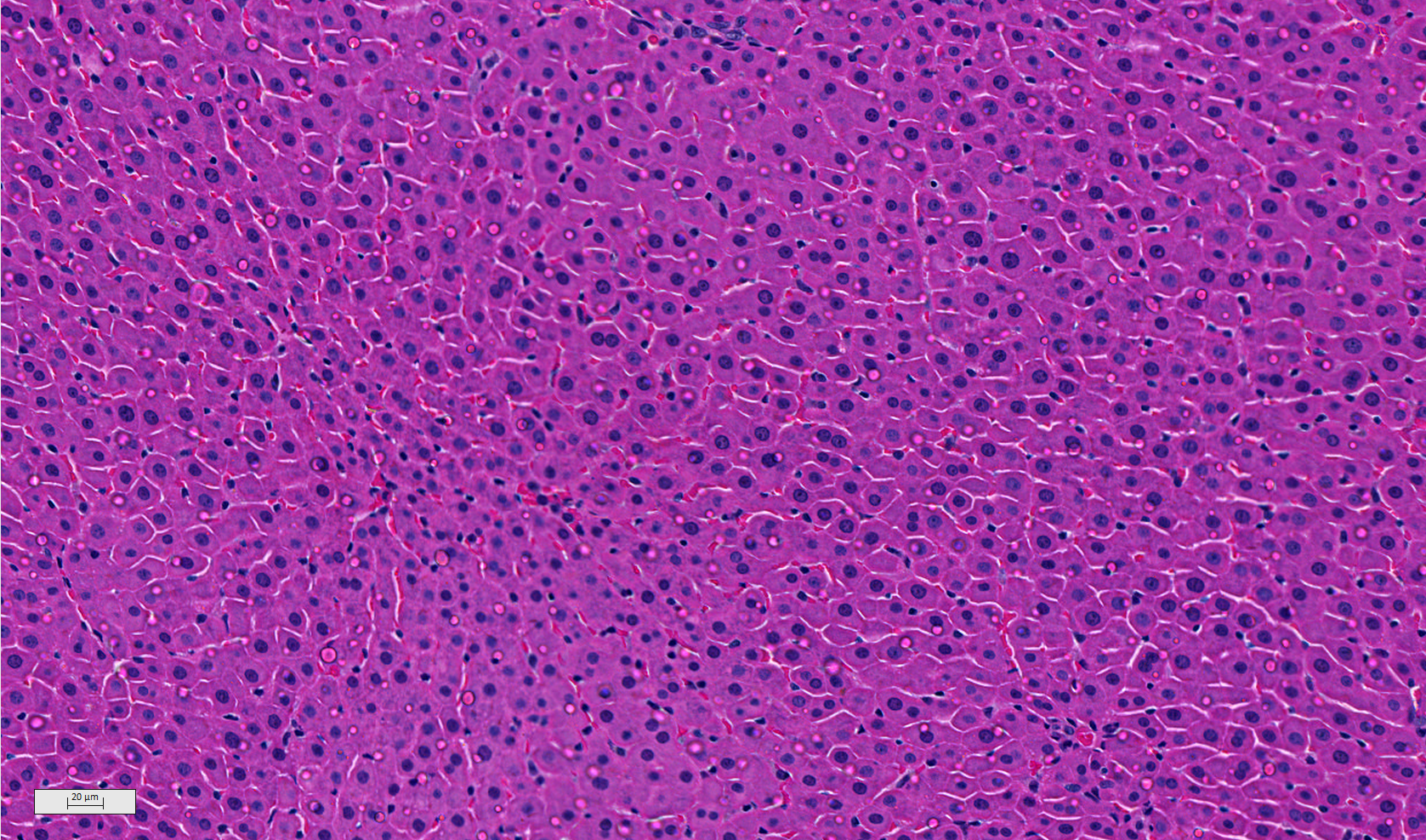

Supplement: Supplementary file 1 [file pharmaceuticals-17-00486-s001.zip › Figure S6 (C). INS.tif]

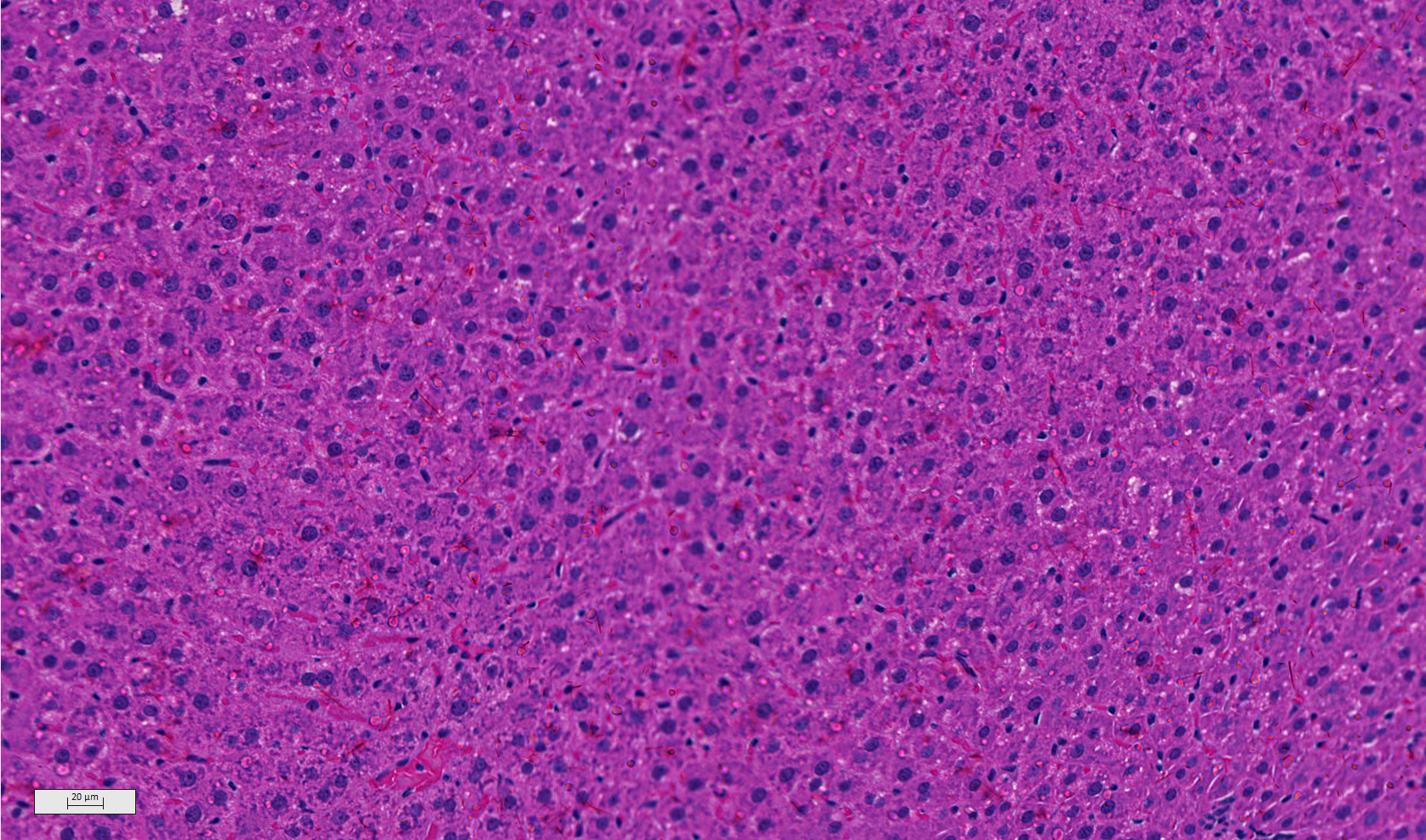

Supplement: Supplementary file 1 [file pharmaceuticals-17-00486-s001.zip › Figure S6 (E). V100.tif]

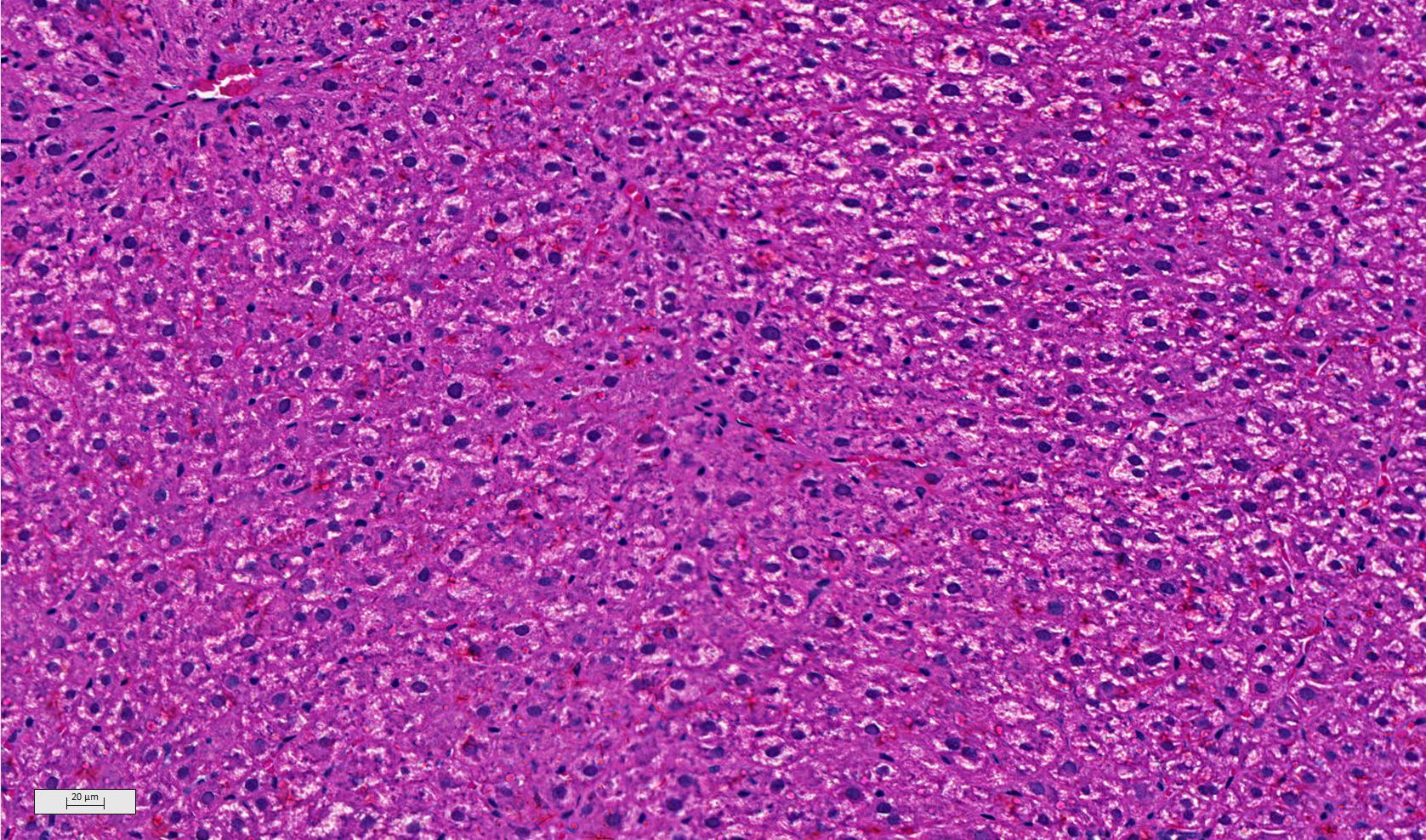

Supplement: Supplementary file 1 [file pharmaceuticals-17-00486-s001.zip › Figure S6 (F). V30INS.tif]

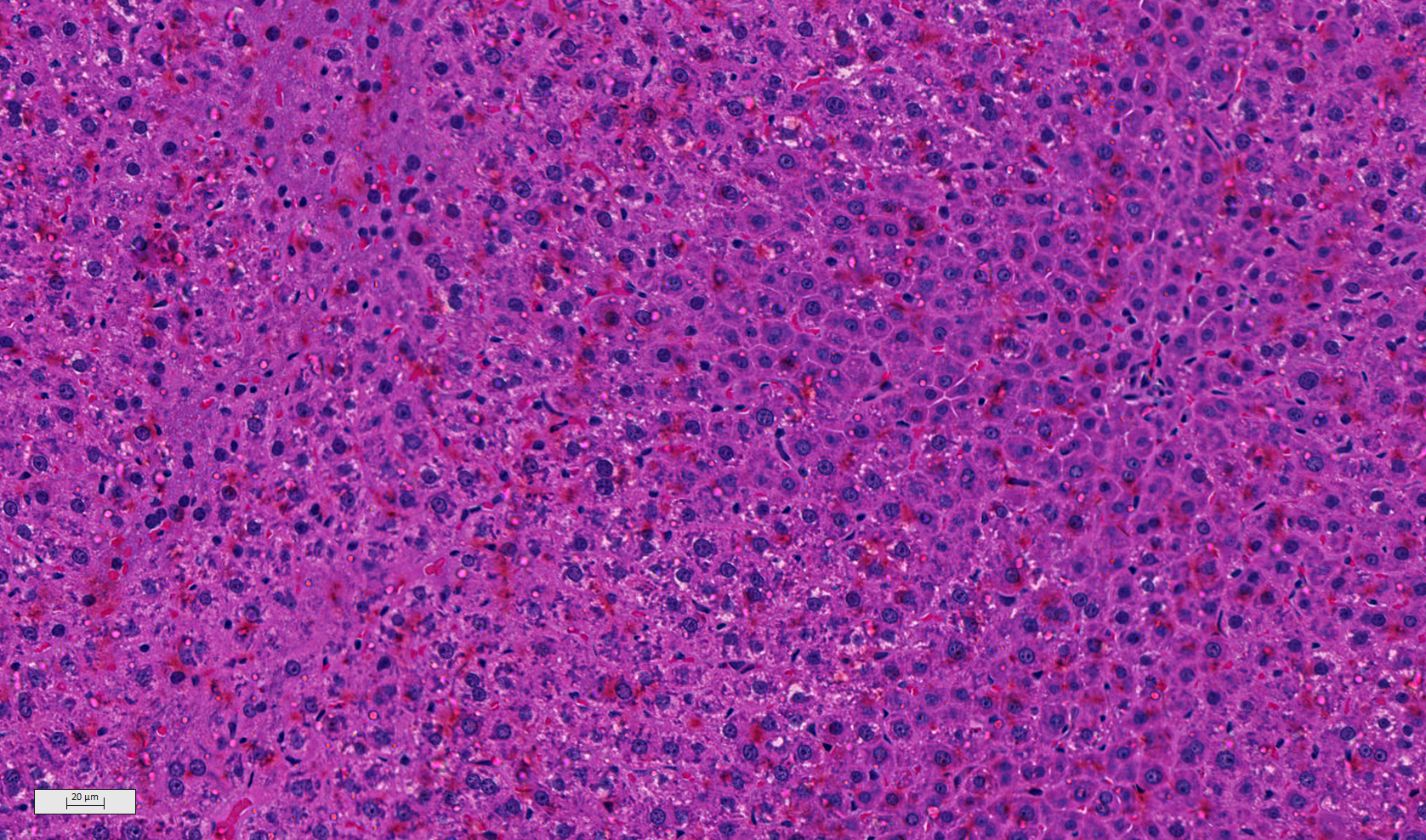

Supplement: Supplementary file 1 [file pharmaceuticals-17-00486-s001.zip › Figure S6 (G). V100INS.tif]

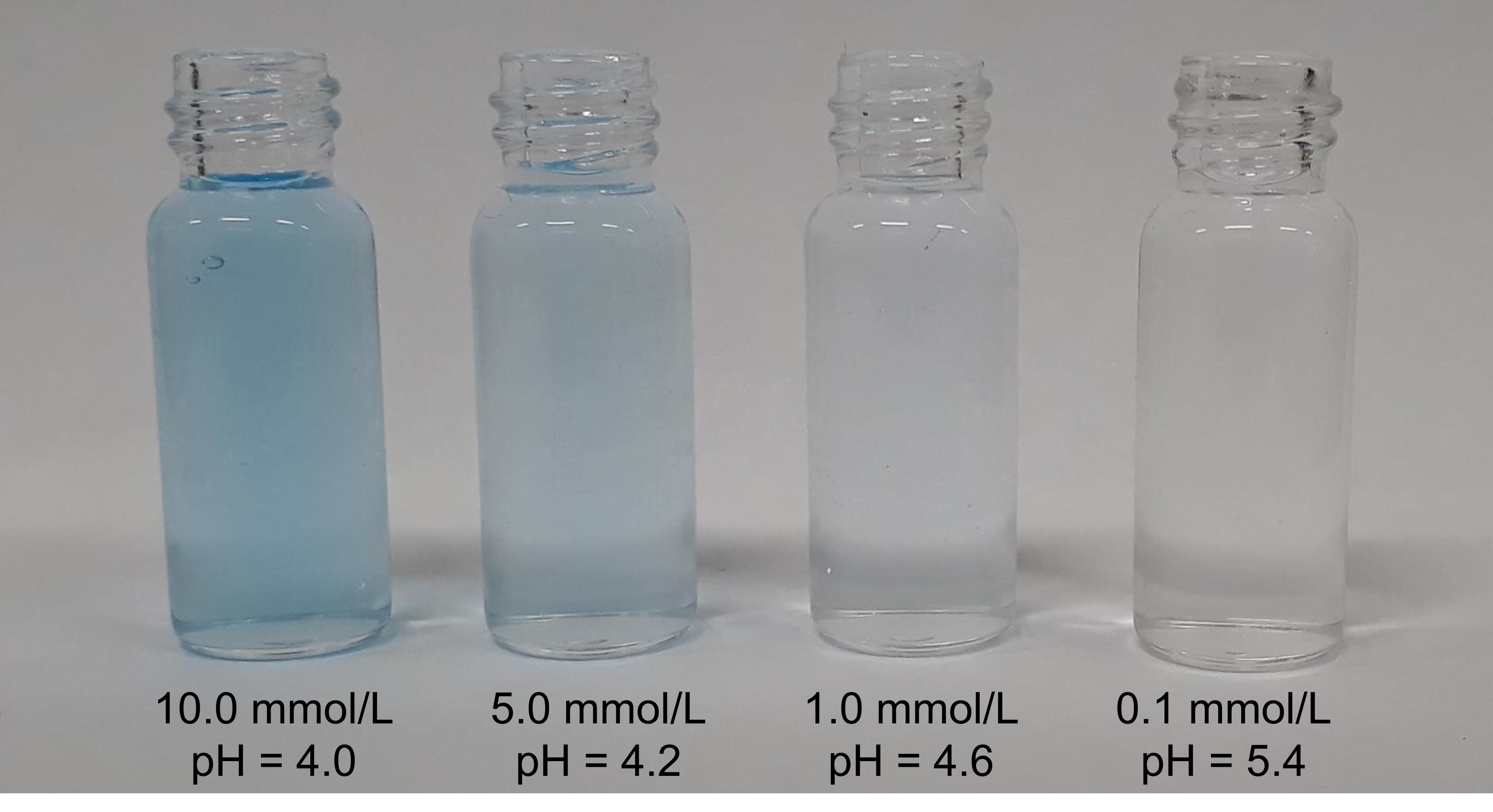

Supplement: Supplementary file 1 [file pharmaceuticals-17-00486-s001.zip › Figure S7. Freshly prepared.jpg]

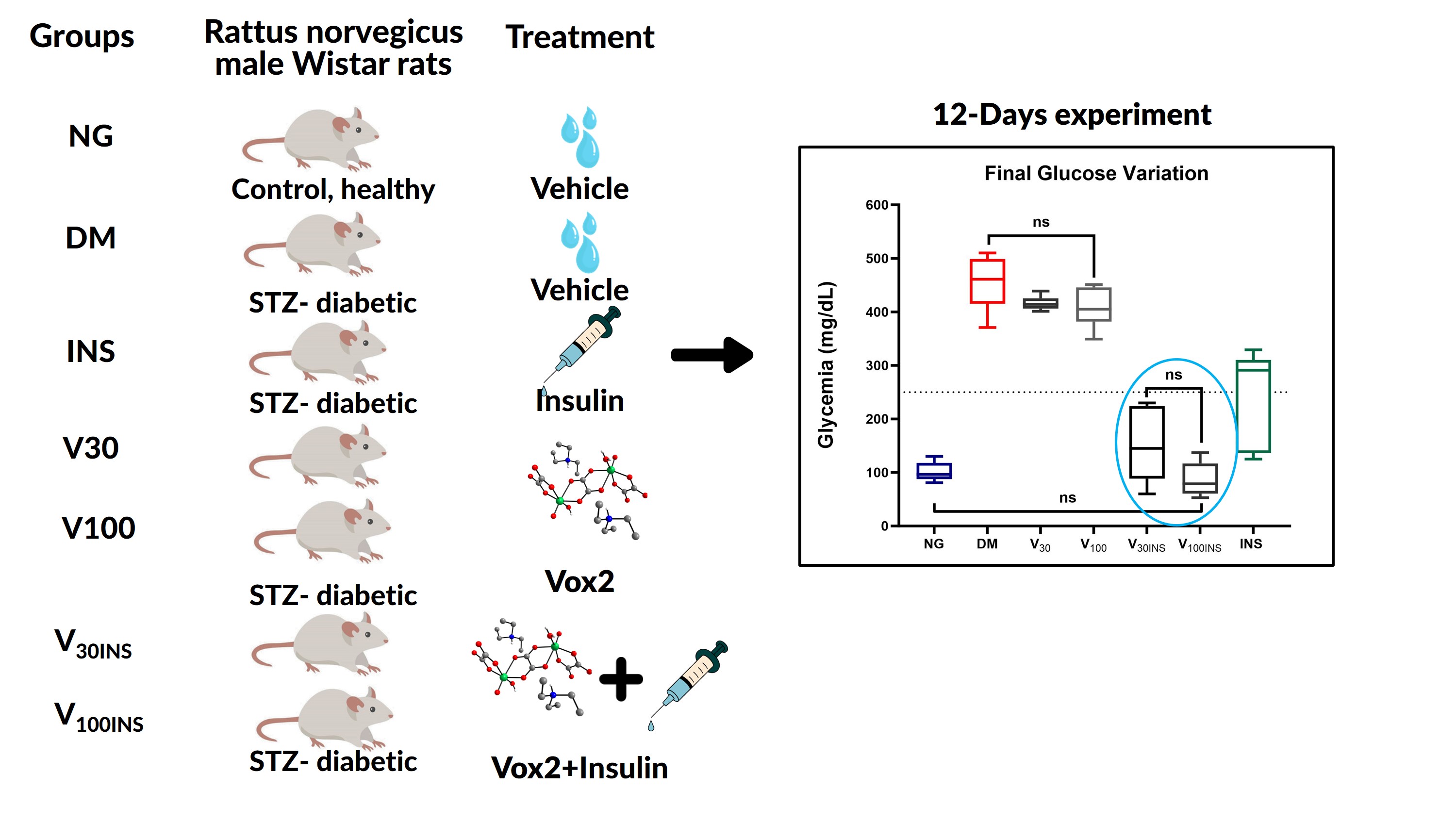

Supplement: Supplementary file 1 [file pharmaceuticals-17-00486-s001.zip › Graphical abstract.jpg]
